# Potential Targets and Action Mechanism of Gastrodin in the Treatment of Attention-Deficit/Hyperactivity Disorder: Bioinformatics and Network Pharmacology Analysis

**DOI:** 10.1155/2022/3607053

**Published:** 2022-09-12

**Authors:** Zhe Song, Guangzhi Luo, Chengen Han, Guangyuan Jia, Baoqing Zhang

**Affiliations:** ^1^The First Clinical Medical College, Shandong University of Traditional Chinese Medicine, Jinan 250355, Shandong, China; ^2^College of Traditional Chinese Medicine, Shandong University of Traditional Chinese Medicine, Jinan 250355, Shandong, China; ^3^Department of Pediatrics, Affiliated Hospital of Shandong University of Traditional Chinese Medicine, Jinan 250011, Shandong, China

## Abstract

**Objective:**

Gastrodin is a main medicinal component of traditional Chinese medicine (TCM) *Gastrodia elata* Blume (*G. elata*), presenting the potential for the treatment of attention-deficit/hyperactivity disorder (ADHD). However, the underlying targets and action mechanisms of the treatment have not been identified.

**Methods:**

The gastrodin-related microarray dataset GSE85871 was obtained from the GEO database and analyzed by GEO2R to obtain differentially expressed genes (DEGs). Subsequently, the targets of gastrodin were supplemented by the Encyclopedia of Traditional Chinese Medicine (ETCM), PubChem, STITCH, and SwissTargetPrediction databases. ADHD-associated genes were collected from six available disease databases (i.e., TTD, DrugBank, OMIM, PharmGKB, GAD, and KEGG DISEASE). The potential targets of gastrodin during ADHD treatment were obtained by mapping gastrodin-related targets with ADHD genes, and their protein–protein interaction (PPI) relationship was constructed by the STRING database. The GO function and KEGG pathway enrichment analyses were performed using the ClueGO plug-in in the Cytoscape software and DAVID database, respectively. Finally, the binding affinity between gastrodin and important targets was verified by molecular docking.

**Results:**

A total of 460 gastrodin-related DEGs were identified from GSE85871, and 124 known gastrodin targets were supplemented from 4 databases, including ETCM. A total of 440 genes were collected from the above 6 disease databases, and 267 ADHD-relevant genes were obtained after duplicate removal. Through mapping the 584 gastrodin targets to the 267 ADHD genes, 16 potential therapeutic targets were obtained, among which the important ones were DRD2, DRD4, CHRNA3, CYP1A1, TNF, IL6, and KCNJ3. The enrichment analysis results indicated that 16 potential targets were involved in 25 biological processes (e.g., dopamine (DA) transport) and 22 molecular functions (e.g., postsynaptic neurotransmitter receptor activity), which were mainly localized at excitatory synapses. The neuroactive ligand-receptor interaction, cholinergic synapse, and dopaminergic synapse might be the core pathways of gastrodin in ADHD treatment. Through molecular docking, it was preliminarily verified that gastrodin showed good binding activity to seven important targets and formed stable binding conformations.

**Conclusions:**

Gastrodin might exert an anti-ADHD effect by upgrading the dopaminergic system and central cholinergic system, inhibiting the inflammatory response and GIRK channel, and exerting a synergistic effect with other drugs on ADHD. For this reason, gastrodin should be considered a multitarget drug for ADHD treatment.

## 1. Introduction

Attention-deficit/hyperactivity disorder (ADHD) is a common neurodevelopmental disorder clinically, mainly characterized by inattention, hyperactivity, and impulsiveness [1]. According to the results of the latest meta-analysis, the global prevalence of ADHD in children and adults was 7.2% and 6.76%, respectively [2, 3], indicating persistent lifetime symptoms in most ADHD patients. Additionally, ADHD is often accompanied by other psychiatric conditions, such as depression disorder, severe anxiety, and oppositional defiant disorder [4]. With the rising incidence in recent years, ADHD has become a global public health concern [5].

ADHD has been defined as a complex, multifactorial disorder, with its etiology and pathogenesis not well understood [6]. Among many hypotheses of ADHD, the abnormalities in the functions of monoamine neurotransmitters have been the research focus; especially, the “dopamine deficit theory” has been confirmed [7, 8]. At present, methylphenidate (MPH) is the most commonly used drug in clinical practice and shows a significant advantage in the temporary control of ADHD symptoms [9]. Unfortunately, MPH is a stimulant that produces potential digestive and cardiovascular side effects. It also causes recurrent symptoms after discontinuation, resulting in poor compliance of patients with treatment [10–12]. Therefore, it is essential to explore a more efficient and safer drug for ADHD treatment.

Considering the clear curative effect, multiple targets, and other advantages of traditional Chinese medicine (TCM) with a long history, Chinese herbs and their main active ingredients have gradually become the major resources of new drugs, represented by artemisinin from *Artemisia annua* [13, 14]. *Gastrodia elata* Blume (*G. elata*) is a high-frequency herb used in the TCM prescription for ADHD treatment [15]. Gastrodin, chemically known as 4-hydroxymethylphenyl *β*-D-glucopyranoside, is the key characteristic medicinal component of *G. elata* [16]. Modern pharmacological studies have revealed that gastrodin has the properties of the regulation of monoamine neurotransmitters [17], anti-inflammation [18], antioxidation [19], antianxiety [20], neuron protection [21], etc., closely related to the known ADHD pathogenesis. Moreover, gastrodin exhibits excellent oral bioavailability, rapid penetration of the blood-brain barrier, and almost nontoxicity [22, 23]. However, the mechanism of gastrodin, as a potential drug for ADHD treatment, on ADHD still remains unclear.

**Figure 1:**
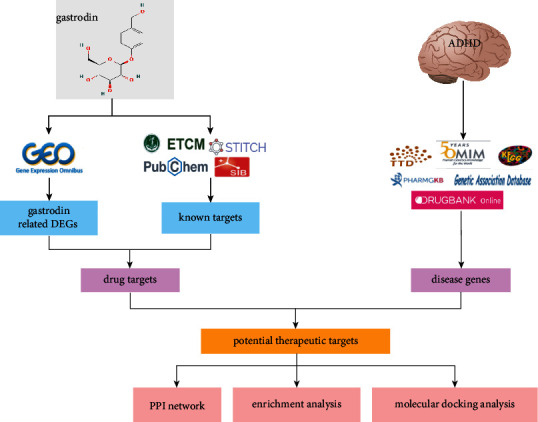
Flowchart of the study on the molecular mechanism of gastrodin in attention-deficit/hyperactivity disorder (ADHD) treatment.

Recent years have seen the emerging bioinformatics and network pharmacology in the field of life science and pharmacology, which offer new methods to elucidate the molecular mechanisms of diseases and drug therapy targets [24]. On this basis, this study was designed to use bioinformatics coupled with network pharmacology to screen and predict the potential targets and signal pathways of gastrodin in ADHD treatment and to verify the predicted results by molecular docking. This study is expected to lay a basis for the follow-up experiment and novel drug development. A flowchart of our study is presented in Figure 1.

## 2. Materials and Methods

### 2.1. Identification of Gastrodin Targets

The microarray dataset GSE85871 was obtained by searching the gene expression omnibus (GEO) database (https://www.ncbi.nlm.nih.gov/geo/) with “gastrodin” as the keyword. The data of GSE85871 were based on the affymetrix human genome U133A 2.0 array (GPL571), containing the gene expression data of 102 TCM ingredient-treated MCF7 cells. Dimethyl sulfoxide (DMSO) treatment was used as a control. Only data from the two gastrodin intervention groups and the two DMSOcontrol groups were extracted and subsequently analyzed. GEO2R (https://www.ncbi.nlm.nih.gov/geo/geo2r/) was used to standardize the raw data and investigate the differentially expressed genes (DEGs). Genes with P<0.01and | fold change (FC)| < 2 were considered DEGs, all of which were displayed by the heatmap and volcano plot.

Afterward, the Encyclopedia of Traditional Chinese Medicine (ETCM) (https://www.tcmip.cn/ETCM/), PubChem (https://pubchem.ncbi.nlm.nih.gov/), STITCH (https://stitch.embl.de/), and SwissTargetPrediction (https://www.swisstargetprediction.ch/) databases were retrieved with “gastrodin” as the keyword, respectively. Then the gastrodin-related drug targets were gained by combining the targets obtained from the above four databases with the DEGs analyzed by the dataset GSE85871.

### 2.2. Collection of ADHD Disease Genes

By retrieving the Therapeutic Target Database (TTD, https://bidd.nus.edu.sg/group/cjttd/), DrugBank (https://go.drugbank.com/), Online Mendelian Inheritance in Man (OMIM, https://www.omim.org/), Pharmacogenomics Knowledge Base (PharmGKB, https://www.pharmgkb.org/), Genetic Association Database (GAD, https://geneticassociationdb.nih.gov/), and KEGG DISEASE (https://www.kegg.jp/kegg/disease/), the ADHD disease genes were collected.

### 2.3. Construction of Protein–Protein Interaction (PPI) Network

The potential targets of gastrodin in ADHD treatment could be obtained by mapping drug targets with disease genes. These potential therapeutic targets were uploaded to the STRING database (https://cn.string-db.org/) with a minimum interaction score of 0.4 to obtain the PPI relationship. Then, they were visualized by the Cytoscape software.

### 2.4. Enrichment Analysis

To further explore the main mechanism of gastrodin in ADHD treatment, the ClueGO plug-in in the Cytoscape software was employed for gene ontology (GO) enrichment analysis, covering biological process (BP), molecular function (MF), and cell composition (CC). Moreover, the Kyoto Encyclopedia of Genes and Genomes (KEGG) pathway enrichment analysis was carried out using the DAVID database (https://david.ncifcrf.gov/), with *P* < 0.05 as the cut-off criterion and the result displayed by the barplot.

### 2.5. Molecular Docking Analysis

The key therapeutic targets were selected for molecular docking with gastrodin to preliminarily verify the mechanism of gastrodin for ADHD treatment. Briefly, the molecular structure of gastrodin was downloaded from the PubChem database, and the format transformation and energy minimization were carried out by the Chem3D software. The obtained structure was imported into the Schrödinger software and saved as the ligand database of molecular docking after hydrogenation, structure optimization, and energy minimization. The crystal structures of the key targets were obtained from the RCSB PDB database (https://www.rcsb.org/) and imported into the Maestro 11.9 platform. The proteins were pretreated by the Protein Preparation Wizard module in Schrödinger software. Constrained energy minimization and geometric structure optimization were performed by the OPLS3e force field. Finally, using the default software parameters, the standard precision (SP) method was selected to dock gastrodin with key therapeutic targets. The docking results were visualized by the PyMOL software.

## 3. Results

### 3.1. Identification of DEGs and Target Collection of Gastrodin

**Figure 2:**
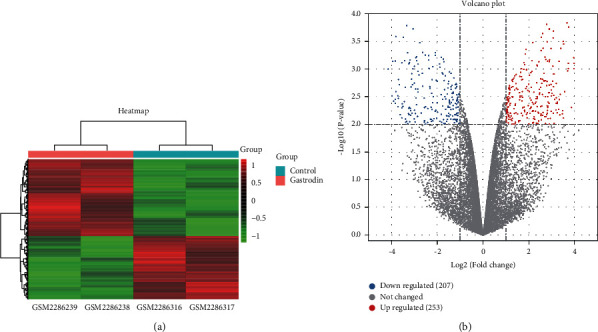
Heatmap and volcano plot of differentially expressed genes (DEGs). (a) Heatmap of 460 DEGs identified with the threshold of *P* < 0.01 and |logFC| > 1. Pink and blue indicate gastrodin intervention groups and DMSO control groups, respectively. The color gradient from red to green represents differential expression values from high to low. Red represents the up-regulated genes, while green denotes the down-regulated genes. (b) Volcano plot of all genes. The red dots indicate up-regulated genes, the blue dots indicate down-regulated genes, and the gray dots indicate genes with no significant difference.

With *P* < 0.01 and |FC| > 2 as the threshold, 460 DEGs (Supplementary Table 1) were identified from the microarray dataset GSE85871, including 253 up-regulated genes and 207 down-regulated genes between the gastrodin intervention groups and control groups. The heatmap and volcano plot were utilized to display the distribution of all DEGs (Figure 2).

By database retrieval, 16, 7, 6, and 103 gastrodin targets (Supplementary Table 2) were obtained in ETCM, PubChem, STITCH, and SwissTargetPrediction, respectively. These targets were combined with 460 DEGs, with the duplicate values deleted. Finally, 584 gastrodin-associated drug targets were obtained (Supplementary Table 3).

### 3.2. Screening of ADHD Disease Genes

A total of 440 known genes of ADHD were collected from 6 existing databases, and 267 ADHD disease genes were obtained after duplicate removal (Supplementary Table 4).

### 3.3. Identification of Therapeutic Targets and Construction of PPI Network

**Figure 3:**
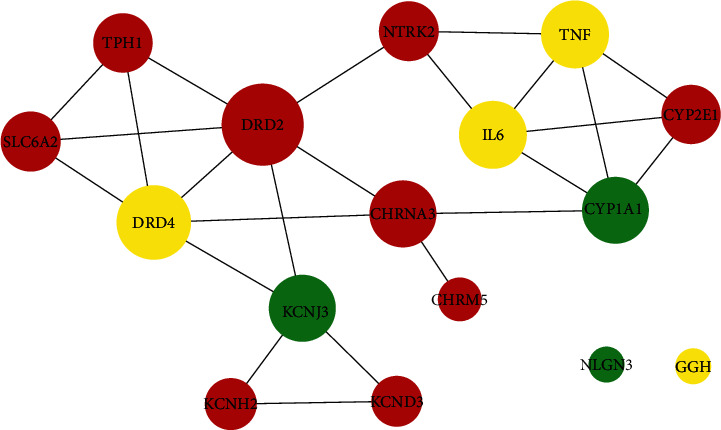
Protein–protein interaction (PPI) network diagram of potential therapeutic targets. Each node in the network represents a target. The node size represents the degree value, and the different color represents differential expression values. Red indicates the up-regulated targets, green indicates the down-regulated targets, yellow indicates targets supplemented by four drug databases, and there is no logFC value.

Among the 584 drug targets and 267 disease genes, 16 common targets were found. By uploading the 16 targets to the STRING database, a PPI network including 14 interactive nodes and 2 independent nodes was obtained and visualized by the Cytoscape software (Table 1 and Figure 3). The NetworkAnalyzer plug-in was employed to evaluate the degree value (i.e., the number of edges linked to a node) of each node. A greater degree value indicates a more significant target in the PPI network and greater biological functions of the target [25]. Only targets with a degree greater than the average value (3.0) were considered important, including DRD2, DRD4, CHRNA3, CYP1A1, TNF, IL6, and KCNJ3.

### 3.4. GO Function and KEGG Pathway Enrichment

**Figure 4:**
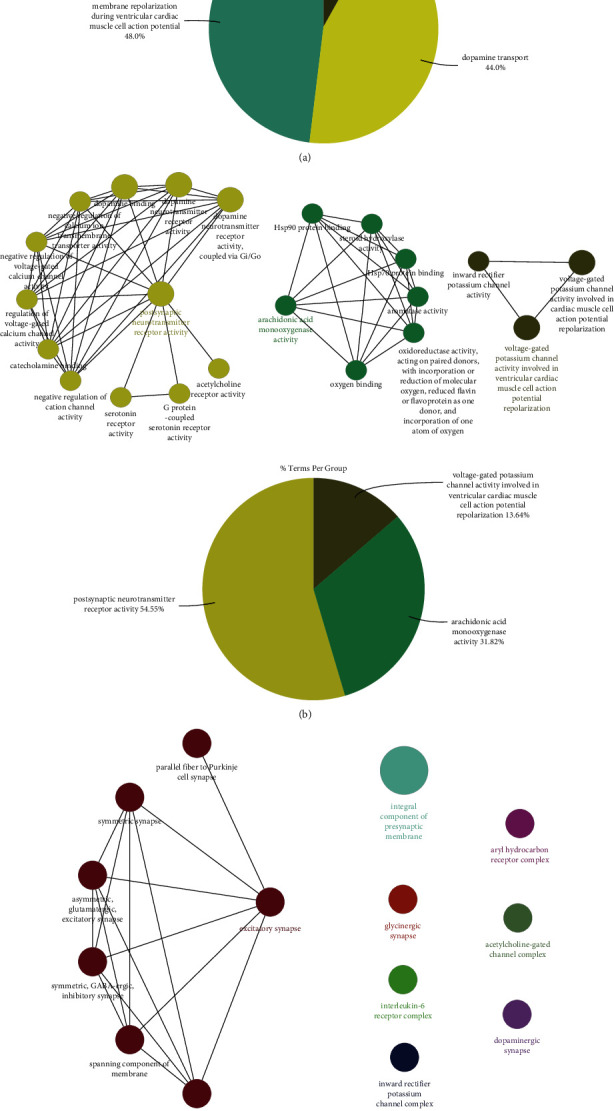
Results of gene ontology (GO) function enrichment analysis. The cluster network and the pie chart of (a) biological process (BP), (b) molecular function (MF), and (c) cell composition (CC). In the cluster network, each node represents a GO term, with the most important term in each group highlighted. The pie chart shows the proportion of each cluster group.

**Figure 5:**
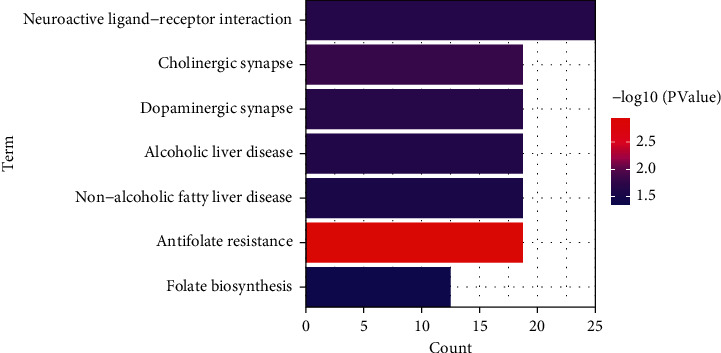
Barplot of the KEGG pathway enrichment. The horizontal axis represents the gene ratio, and the vertical axis represents the KEGG pathways. The color gradient of bars from red to blue indicates the (*P*) values from low to high.

The ClueGO plug-in in the Cytoscape software was utilized to perform the enrichment analysis of BP, MF, and CC on the above 16 targets. As shown in Figure 4(a), 25 biological processes were obtained and clustered into 3 groups: membrane repolarization during ventricular cardiac muscle cell action potential, dopamine (DA) transport, and response to nicotine. Among the three groups, DA transport had close ties to the pathological mechanism of ADHD, covering 44.0% of all BP terms. As can be seen in Figure 4(b), 16 targets mediated 22 molecular functions, which were also clustered into three groups, i.e., the postsynaptic neurotransmitter receptor activity, arachidonic acid monooxygenase activity, and voltage-gated potassium channel activity involved in ventricular cardiac muscle cell action potential repolarization. Among them, the postsynaptic neurotransmitter receptor activity was the main enriched molecular function, covering 54.55% of all MF terms. The CC result demonstrated that the potential targets of gastrodin during ADHD treatment were located on 14 cell compositions clustered into 8 groups (Figure 4(c)). Among them, the excitatory synapse was the main enriched area (covering 50%). The KEGG pathway enrichment analysis indicated 16 targets associated with 7 pathways (*P* < 0.05), mainly including the neuroactive ligand-receptor interaction, cholinergic synapse, and dopaminergic synapse (Figure 5).

### 3.5. Molecular Docking of Gastrodin with Important Therapeutic Targets

**Figure 6:**
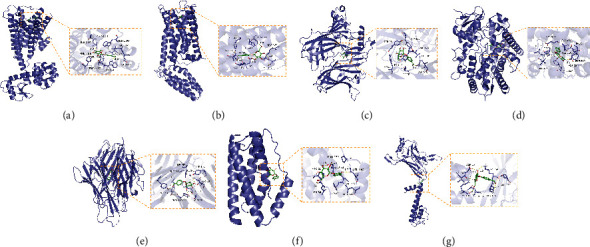
Docking model of gastrodin with seven important targets. (a) DRD2 and gastrodin; (b) DRD4 and gastrodin; (c) CHRNA3 and gastrodin; (d) CYP1A1 and gastrodin; (e) TNF and gastrodin; (f) IL6 and gastrodin; (g) KCNJ3 and gastrodin.

Gastrodin was docked with DRD2, DRD4, CHRNA3, CYP1A1, TNF, IL6, and KCNJ3, respectively, to evaluate the binding affinity between gastrodin and these target proteins. Generally, binding energy less than 0 indicates that the ligand can bind to the receptor spontaneously [26]; binding energy less than −5.00 kcal/mol implies strong binding activity [27]. As can be seen from Table 2, the binding energies of gastrodin and seven important targets were all less than −6.00 kcal/mol. As shown in Figure 6, gastrodin could form stable complexes with seven target proteins mainly through the formation of hydrogen bonds or hydrophobic interaction. In short, the docking results provide data support for the subsequent verification of the regulatory relationship between gastrodin and these targets.

## 4. Discussion

ADHD was previously considered a behavioral disorder only in childhood, with the symptoms disappearing with age. Nevertheless, growing evidence has revealed that more than half of patients will continue to suffer from ADHD until adulthood and even throughout life [28, 29]. ADHD leads to declining academic performance, cognitive impairment, various emotional problems, and increasing social crime and suicide rates [30, 31], resulting in significant negative consequences for individual patients, their families, and society [32]. Due to the complex etiology and pathogenesis, it is difficult to eliminate the symptoms using the existing therapeutic drugs, which have many side effects and thus pose severe challenges to clinical treatment [33]. Fortunately, Chinese herbs and their active ingredients bring new opportunities for drug research and development, represented by a frequently used herb *G. elata* in ADHD treatment, with gastrodin as its key component [34]. Therefore, elucidation of the specific mechanism of gastrodin during ADHD treatment can provide guidance for new drug development and clinical treatment.

This study systematically analyzed potential targets and signal pathways of gastrodin during ADHD treatment. By retrieving the GEO database, several drug databases, and disease databases, 16 potential therapeutic targets were identified, among which the important were DRD2, DRD4, CHRNA3, CYP1A1, TNF, IL6, and KCNJ3. Both DRD2 and DRD4 belonged to DA D2-like receptors, participating in DA transmission [35]. DA is a key neurotransmitter regulating physical movement, emotion, and neuroendocrine activities [36], stored in the synaptic vesicles of dopaminergic neurons after synthesis. When nerve impulses are afferent, DA is released into the synaptic cleft and binds to DA receptors to perform multiple functions [37]. A genome-wide association study of first-line pharmacotherapeutics for ADHD suggested that DRD2 might be a secondary target of MPH and amphetamine (AMP) [38]. Decreased DRD2 expression could lead to hyperactive behavior in mice [39]. A neuroimaging experiment has also confirmed that the decreasingly available DA D2 autoreceptors were closely related to impulsive traits [40]. According to our results (Table 1 and Figure 3), gastrodin might promote DRD2 expression, suggesting the potential ameliorative effects of gastrodin on the impulsive and hyperactive symptoms of ADHD by increasing the number of DRD2 or its sensitivity to DA. Regarded as a therapeutic target for ADHD [41], DRD4 represented a lower density in multiple brain regions of ADHD patients than that of normal individuals [42, 43]. The application of highly selective DRD4 agonists could significantly improve the cognitive ability of ADHD model rats; moreover, it did not increase the risk of substance abuse compared with psychostimulant therapy [44, 45]. To our knowledge, the targeting relationship between gastrodin and DRD4 has not been reported. Our molecular docking results showed a strong binding affinity of gastrodin with DRD4 (binding energy: −7.13 kcal/mol). As shown in Figure 6(b), gastrodin could interact with multiple amino acid residues of DRD4 through hydrophobic interaction and hydrogen bonding. Therefore, DRD4 might become a new target for gastrodin in disease treatment. Particularly in ADHD treatment, gastrodin might play a role similar to that of a DRD4 agonist. CHRNA3 is a member of the nicotinic acetylcholine receptor (nAChR) family, and nAChR dysfunction plays an important role in the pathological mechanism of attention deficit [46]. Several novel nAChR agonists have been developed, such as AZD3480, ABT-894, and ABT-089. They can improve adult ADHD symptoms to a certain extent according to the randomized controlled phase II clinical trials [47–49], indicating the feasibility of using gastrodin to treat ADHD by increasing CHRNA3 expression. CYP1A1 is an important paralog of CYP1A2, both of which are members of the cytochrome P450 (CYP450) superfamily [50]. CYP450 is among the most critical drug metabolic enzymes capable of catalyzing phase I reactions of drugs [51]. AMP, atomoxetine, and tricyclic antidepressants, commonly used in ADHD treatment, are metabolized by CYP1A2 [52], but their relationship with CYP1A1 has not been reported. Our results suggested that gastrodin might down-regulate CYP1A1 expression. It was speculated that a beneficial interaction existed between the CYP450-metabolized therapeutic drugs for ADHD and gastrodin. Specifically, gastrodin might reduce the metabolic rate of these drugs *in vivo* by inhibiting CYP1A1 activity, thereby improving their efficacy. TNF and IL6 are well-known important cytokines of inflammation and immune response. Excess inflammatory cytokines could influence the turnover of monoamine neurotransmitters and induce various neuropsychiatric disorders [53]. Inflammation has been implicated as a trigger of ADHD [54], and gastrodin has been proved to have anti-inflammatory pharmacological effects, which can alleviate cognitive impairment by lessening TNF-*α* and IL-6 levels [18]. KCNJ3, also termed GIRK1 or Kir3.1, is extensively expressed in the central nervous system and binds to three other potassium channel proteins (i.e., KCNJ6/GIRK2, KCNJ9/GIRK3, and KCNJ5/GIRK4) to form the G protein-gated inwardly rectifying potassium (GIRK) channel [55, 56]. The GIRK channel can bind to DA D2-like receptors, resulting in neuron self-inhibition and reduced DA release [57]. This is relevant to the pathogenesis of many neuropsychiatric disorders, such as ADHD, schizophrenia, and epilepsy [58]. GIRK channel blockers could prevent drug-induced hyperactivity in mice [59]. In our study, gastrodin might down-regulate KCNJ3 expression, implying that inhibition of the GIRK channel through KCNJ3 might be a main mechanism of gastrodin during ADHD treatment.

Subsequently, further functional analysis was conducted on the potential targets of gastrodin in ADHD treatment. As shown in Figure 4, these targets mainly participated in the biological process of DA transport and the molecular function of postsynaptic neurotransmitter receptor activity, mainly located at excitatory synapses. The KEGG pathway enrichment analysis results highlighted that the neuroactive ligand-receptor interaction, cholinergic synapse, and dopaminergic synapse were the main pathways for the gastrodin therapeutic effect (Figure 5). According to the results obtained in this study, the important targets and pathways of gastrodin in ADHD treatment were closely related to DA receptor activity, nAChR activity, drug interaction, inflammatory response, GIRK channel, and neurotransmitter transmission. Therefore, the specific mechanisms of gastrodin in ADHD treatment could be concluded from the following aspects: (1) Above all, gastrodin might promote the release and transport of DA by enhancing the function of DA receptors, as well as inhibiting proinflammatory cytokines and GIRK channel. (2) Gastrodin elevated the function of the central cholinergic system by acting on nAChR. (3) Gastrodin might reduce the metabolic rate of CYP450-metabolized therapeutic drugs for ADHD, and the combination of gastrodin with these drugs might have a synergistic effect.

Our study also has some limitations. First of all, the sample size in the gastrodin intervention group was poor in dataset GSE85871, which took MCF7 cells as the object to analyze the gene expression of different TCM ingredients. The results would definitely vary if different human cells and tissues were used for research. Second, the FC value of gastrodin targets supplemented by the four drug databases could not be obtained, and the exact effects of gastrodin on the predicted targets and pathways need further experimental verification. Finally, distinguishment was not made between the ADHD subtypes. In accordance with the Diagnostic and Statistical Manual of Mental Disorders, 5th edition (DSM-5), ADHD could be classified as hyperactive/impulsive type, inattentive type, and combined type [60], which presented diverse clinical manifestations and pathogenesis [61]. In the future, based on the results of this study, the effective mechanisms of gastrodin in ADHD treatment will be first verified. Then, detailed clinical and experimental research programs are expected to be designed to observe the effects of gastrodin on different subtypes of ADHD.

## 5. Conclusions

In conclusion, according to bioinformatics and network pharmacology studies, the main targets of gastrodin for ADHD treatment might be DRD2, DRD4, CHRNA3, CYP1A1, TNF, IL6, and KCNJ3, which were preliminarily verified by molecular docking. Gastrodin might exert an anti-ADHD effect by enhancing the function of the dopaminergic system and central cholinergic system, inhibiting the inflammatory response and GIRK channel, and exerting a synergistic effect with other drugs on ADHD. Among them, gastrodin might promote the release and transport of DA through various mechanisms, which deserves the most attention. Gastrodin should be considered a multitarget drug for ADHD treatment. Taken together, the present study is expected to provide important information to further complement the pharmacological effects of gastrodin and the clinical ADHD treatment.

## Figures and Tables

**Table 1:** Basic information of 16 potential therapeutic targets.

No.	Gene symbol	Gene name	Degree	logFC
1	DRD2	Dopamine receptor D2	6	2.4680278
2	DRD4	Dopamine receptor D4	5	—
3	CHRNA3	Cholinergic receptor nicotinic alpha 3 subunit	4	2.5349484
4	CYP1A1	Cytochrome P450 family 1 subfamily A, member 1	4	−3.8387415
5	TNF	Tumor necrosis factor	4	—
6	IL6	Interleukin 6	4	—
7	KCNJ3	Potassium inwardly rectifying channel, subfamily J, member 3	4	−1.1164583
8	CYP2E1	Cytochrome P450 family 2, subfamily E, member 1	3	2.7216362
9	SLC6A2	Solute carrier family 6, member 2	3	2.5546023
10	TPH1	Tryptophan hydroxylase 1	3	2.1948648
11	NTRK2	Neurotrophic receptor tyrosine kinase 2	3	2.7099514
12	KCND3	Potassium voltage-gated channel subfamily D, member 3	2	3.0669291
13	KCNH2	Potassium voltage-gated channel subfamily H, member 2	2	1.127879
14	CHRM5	Cholinergic receptor muscarinic 5	1	1.8235883
15	GGH	Gamma-glutamyl hydrolase	0	—
16	NLGN3	Neuroligin 3	0	−1.8037092

**Table 2:** The result of molecular docking.

Target	PDB ID	Binding energy (kcal/mol)
DRD2	6CM4	−7.46
DRD4	5WIV	−7.13
CHRNA3	4ZK4	−7.93
CYP1A1	4I8V	−7.33
TNF	7KPA	−8.35
IL6	4O9H	−6.64
KCNJ3	2QKS	−6.52

## Data Availability

The data supporting the conclusions of this article are included within the article.

## Supplementary Materials

Supplementary Materials Supplementary Table 1: the 460 DEGs in GSE85871. Supplementary Table 2: the known targets of gastrodin in four drug databases. Supplementary Table 3: 584 gastrodin-related drug targets. Supplementary Table 4: the ADHD-related disease genes.
