# Supplementary material for: Potential Targets and Action Mechanism of Gastrodin in the Treatment of Attention-Deficit/Hyperactivity Disorder: Bioinformatics and Network Pharmacology Analysis
Source: Evid Based Complement Alternat Med. 2022 Sep 12;2022:3607053. doi: 10.1155/2022/3607053 (PMC9484880; doi:10.1155/2022/3607053)
Supplement: Supplementary Materials — Supplementary Table 1: the 460 DEGs in GSE85871. Supplementary Table 2: the known targets of gastrodin in four drug databases. Supplementary Table 3: 584 gastrodin-related drug targets. Supplementary Table 4: the ADHD-related disease genes. [file 3607053.f1.zip › 3607053.f1/Supplementary Table 1.docx]

| ID | P.Value | logFC | Gene.symbol | Gene.title |
| --- | --- | --- | --- | --- |
| 206702_at | 0.001001 | -4.1503971 | TEK | TEK receptor tyrosine kinase |
| 216364_s_at | 0.000792 | 3.9886431 | AFF2 | AF4/FMR2 family member 2 |
| 205018_s_at | 0.000981 | 3.981872 | MBNL2 | muscleblind like splicing regulator 2 |
| 209396_s_at | 0.003063 | -3.9809898 | CHI3L1 | chitinase 3 like 1 |
| 207341_at | 0.000625 | 3.9706594 | PRTN3 | proteinase 3 |
| 220095_at | 0.000726 | -3.9456471 | CNTLN | centlein |
| 208274_at | 0.003822 | -3.9079787 | OCLM | oculomedin |
| 208375_at | 0.004994 | 3.87293 | IFNA1 | interferon alpha 1 |
| 205749_at | 0.000717 | -3.8387415 | CYP1A1 | cytochrome P450 family 1 subfamily A member 1 |
| 215564_at | 0.003529 | -3.8370153 | AREG | amphiregulin |
| 216191_s_at | 0.004209 | -3.8327511 | TRDV3 | T cell receptor delta variable 3 |
| 211122_s_at | 0.000257 | -3.8116884 | CXCL11 | C-X-C motif chemokine ligand 11 |
| 217146_at | 0.002574 | -3.8108899 | JRK | Jrk helix-turn-helix protein |
| 203471_s_at | 0.000176 | 3.7493541 | PLEK | pleckstrin |
| 220972_s_at | 0.000789 | 3.7338307 | KRTAP9-9 | keratin associated protein 9-9 |
| 221057_at | 0.000981 | 3.732744 | SPATA1 | spermatogenesis associated 1 |
| 215104_at | 0.003057 | -3.7246701 | NRIP2 | nuclear receptor interacting protein 2 |
| 206381_at | 0.0048 | -3.7197991 | SCN2A | sodium voltage-gated channel alpha subunit 2 |
| 208288_at | 0.001084 | 3.7058727 | ABCB11 | ATP binding cassette subfamily B member 11 |
| 220759_at | 0.003397 | -3.7039476 | EDDM3B | epididymal protein 3B |
| 216839_at | 0.001556 | -3.7003508 | LAMA2 | laminin subunit alpha 2 |
| 220269_at | 0.000148 | 3.6755121 | ZBBX | zinc finger B-box domain containing |
| 213170_at | 0.001922 | -3.6620623 | GPX7 | glutathione peroxidase 7 |
| 214267_s_at | 0.006711 | -3.6208393 | CADM4 | cell adhesion molecule 4 |
| 215289_at | 0.002576 | 3.571398 | ZNF749 | zinc finger protein 749 |
| 203681_at | 0.007689 | -3.5503612 | IVD | isovaleryl-CoA dehydrogenase |
| 219707_at | 0.000772 | -3.5502981 | CPNE7 | copine 7 |
| 210729_at | 0.005042 | -3.5481798 | NPY2R | neuropeptide Y receptor Y2 |
| 220978_at | 0.004275 | -3.5308687 | KRTAP1-3 | keratin associated protein 1-3 |
| 219812_at | 0.000352 | 3.5284434 | PVRIG | poliovirus receptor related immunoglobulin domain containing |
| 221631_at | 0.007732 | 3.5217593 | CACNA1I | calcium voltage-gated channel subunit alpha1 I |
| 221910_at | 0.000317 | 3.5067026 | ETV1 | ETS variant 1 |
| 216865_at | 0.004747 | -3.5014194 | COL14A1 | collagen type XIV alpha 1 chain |
| 212077_at | 0.005715 | 3.4888474 | CALD1 | caldesmon 1 |
| 207216_at | 0.000265 | -3.4825339 | TNFSF8 | tumor necrosis factor superfamily member 8 |
| 214326_x_at | 0.006503 | 3.4710756 | JUND | JunD proto-oncogene, AP-1 transcription factor subunit |
| 207033_at | 0.003906 | -3.4403254 | GIF | gastric intrinsic factor |
| 217093_at | 0.000706 | -3.4223383 | RNASE1 | ribonuclease A family member 1, pancreatic |
| 215770_at | 0.000422 | 3.4050418 | OR7E2P | olfactory receptor family 7 subfamily E member 2 pseudogene |
| 212125_at | 0.000422 | 3.4020004 | RANGAP1 | Ran GTPase activating protein 1 |
| 213479_at | 0.002139 | -3.4015351 | NPTX2 | neuronal pentraxin 2 |
| 207815_at | 0.008066 | -3.3853596 | PF4V1 | platelet factor 4 variant 1 |
| 208204_s_at | 0.009186 | -3.3793521 | CAV3 | caveolin 3 |
| 206112_at | 0.002875 | 3.3788397 | ANKRD7 | ankyrin repeat domain 7 |
| 217395_at | 0.006795 | 3.3609502 | MT4 | metallothionein 4 |
| 207177_at | 0.008902 | -3.3377424 | PTGFR | prostaglandin F receptor |
| 220834_at | 0.000166 | -3.3357491 | MS4A12 | membrane spanning 4-domains A12 |
| 220911_s_at | 0.003587 | 3.334154 | NYNRIN | NYN domain and retroviral integrase containing |
| 205155_s_at | 0.000726 | 3.3260644 | SPTBN2 | spectrin beta, non-erythrocytic 2 |
| 202270_at | 0.005385 | 3.3101466 | GBP1 | guanylate binding protein 1 |
| 208010_s_at | 0.000508 | -3.2969628 | PTPN22 | protein tyrosine phosphatase, non-receptor type 22 |
| 221801_x_at | 0.009783 | -3.2904171 | NEFL | neurofilament, light polypeptide |
| 219463_at | 0.006437 | -3.2619316 | LAMP5 | lysosomal associated membrane protein family member 5 |
| 201283_s_at | 0.00422 | 3.2481359 | TRAK1 | trafficking kinesin protein 1 |
| 211054_at | 0.004002 | 3.2467416 | INVS | inversin |
| 210507_s_at | 0.00515 | -3.2447149 | AVIL | advillin |
| 206260_at | 0.006225 | 3.2434565 | TGM4 | transglutaminase 4 |
| 207817_at | 0.000803 | -3.2402889 | IFNW1 | interferon omega 1 |
| 220210_at | 0.00674 | -3.2335867 | CHRNA10 | cholinergic receptor nicotinic alpha 10 subunit |
| 206984_s_at | 0.004946 | -3.2142135 | RIT2 | Ras like without CAAX 2 |
| 222226_at | 0.000762 | -3.2137316 | SAA3P | serum amyloid A3 pseudogene |
| 220343_at | 0.003326 | 3.207707 | PDE7B | phosphodiesterase 7B |
| 210814_at | 0.001832 | 3.204544 | TRPC3 | transient receptor potential cation channel subfamily C member 3 |
| 33767_at | 0.009051 | -3.2045377 | NEFH | neurofilament heavy polypeptide |
| 214569_at | 0.004173 | 3.2040875 | IFNA5 | interferon alpha 5 |
| 214575_s_at | 0.005516 | 3.1914627 | AZU1 | azurocidin 1 |
| 217183_at | 0.000686 | -3.1863429 | SPC24 | SPC24, NDC80 kinetochore complex component |
| 210432_s_at | 0.005175 | -3.1779569 | SCN3A | sodium voltage-gated channel alpha subunit 3 |
| 214131_at | 0.005669 | -3.1641071 | TXLNGY | taxilin gamma pseudogene, Y-linked |
| 216845_x_at | 0.006859 | -3.1589998 | KMT2D | lysine methyltransferase 2D |
| 214625_s_at | 0.002116 | -3.1457125 | MINK1 | misshapen like kinase 1 |
| 205158_at | 0.003569 | 3.1352079 | RNASE4 | ribonuclease A family member 4 |
| 210227_at | 0.001524 | -3.1224212 | DLGAP2 | DLG associated protein 2 |
| 220271_x_at | 0.009648 | 3.1139537 | EFCAB6 | EF-hand calcium binding domain 6 |
| 210202_s_at | 0.001235 | 3.1133171 | BIN1 | bridging integrator 1 |
| 221426_s_at | 0.001215 | -3.1131796 | OR3A3 | olfactory receptor family 3 subfamily A member 3 |
| 219885_at | 0.006308 | -3.101682 | SLFN12 | schlafen family member 12 |
| 221961_at | 0.003008 | 3.0907548 | CLCN7 | chloride voltage-gated channel 7 |
| 211151_x_at | 0.003101 | -3.0901387 | GH1 | growth hormone 1 |
| 220403_s_at | 0.001921 | -3.0857475 | TP53AIP1 | tumor protein p53 regulated apoptosis inducing protein 1 |
| 207323_s_at | 0.002099 | 3.085615 | MBP | myelin basic protein |
| 219011_at | 0.000208 | 3.0828242 | PLEKHA4 | pleckstrin homology domain containing A4 |
| 206259_at | 0.001227 | 3.0821273 | PROC | protein C, inactivator of coagulation factors Va and VIIIa |
| 214254_at | 0.006311 | 3.0706852 | MAGEA4 | MAGE family member A4 |
| 215014_at | 0.000273 | 3.0669291 | KCND3 | potassium voltage-gated channel subfamily D member 3 |
| 206973_at | 0.000994 | -3.0628795 | PPFIA2 | PTPRF interacting protein alpha 2 |
| 213436_at | 0.004349 | 3.0594713 | CNR1 | cannabinoid receptor 1 |
| 211128_at | 0.00019 | -3.0579477 | EDA | ectodysplasin A |
| 207327_at | 0.000888 | 3.0559842 | EYA4 | EYA transcriptional coactivator and phosphatase 4 |
| 206242_at | 0.001657 | 3.0474459 | TM4SF5 | transmembrane 4 L six family member 5 |
| 209347_s_at | 0.002763 | 3.0190306 | MAF | MAF bZIP transcription factor |
| 221366_at | 0.008033 | -3.0082206 | NKX6-1 | NK6 homeobox 1 |
| 214120_at | 0.001799 | -3.0042574 | RFPL1S | RFPL1 antisense RNA 1 |
| 205979_at | 0.000892 | -3.0007271 | SCGB2A1 | secretoglobin family 2A member 1 |
| 208981_at | 0.005578 | -2.9987929 | PECAM1 | platelet and endothelial cell adhesion molecule 1 |
| 215627_at | 0.002414 | 2.9883917 | LOC101926913 | uncharacterized LOC101926913 |
| 206699_x_at | 0.003068 | 2.9869439 | NPAS1 | neuronal PAS domain protein 1 |
| 205943_at | 0.005831 | 2.9789862 | TDO2 | tryptophan 2,3-dioxygenase |
| 214868_at | 0.000423 | 2.9742879 | PIWIL1 | piwi like RNA-mediated gene silencing 1 |
| 214708_at | 0.001901 | 2.9739577 | SNTB1 | syntrophin beta 1 |
| 206207_at | 0.009095 | -2.9691673 | CLC | Charcot-Leyden crystal galectin |
| 214444_s_at | 0.006425 | -2.9533686 | PVR | poliovirus receptor |
| 216493_s_at | 0.002303 | -2.9438946 | IGF2BP3 | insulin like growth factor 2 mRNA binding protein 3 |
| 202803_s_at | 0.007838 | 2.9411349 | ITGB2 | integrin subunit beta 2 |
| 207795_s_at | 0.000623 | -2.935268 | KLRD1 | killer cell lectin like receptor D1 |
| 209960_at | 0.003368 | -2.9292428 | HGF | hepatocyte growth factor |
| 205944_s_at | 0.000682 | 2.9106817 | CLTCL1 | clathrin heavy chain like 1 |
| 218232_at | 0.00444 | -2.9098517 | C1QA | complement C1q A chain |
| 210585_s_at | 0.00019 | 2.9083397 | TNPO2 | transportin 2 |
| 210339_s_at | 0.0006 | -2.9047994 | KLK2 | kallikrein related peptidase 2 |
| 210934_at | 0.009891 | 2.9017054 | BLK | BLK proto-oncogene, Src family tyrosine kinase |
| 202316_x_at | 0.002677 | 2.9016696 | UBE4B | ubiquitination factor E4B |
| 201249_at | 0.000735 | 2.9000408 | SLC2A1 | solute carrier family 2 member 1 |
| 217174_s_at | 0.000708 | -2.8948455 | APC2 | APC2, WNT signaling pathway regulator |
| 220793_at | 0.001957 | 2.8944212 | SAGE1 | sarcoma antigen 1 |
| 221054_s_at | 0.000193 | 2.8920527 | TCL6 | T-cell leukemia/lymphoma 6 (non-protein coding) |
| 205269_at | 0.004168 | 2.8872164 | LCP2 | lymphocyte cytosolic protein 2 |
| 205614_x_at | 0.004528 | 2.8762491 | MST1 | macrophage stimulating 1 |
| 205468_s_at | 0.007253 | -2.8742216 | IRF5 | interferon regulatory factor 5 |
| 212822_at | 0.002688 | 2.8643889 | HEG1 | heart development protein with EGF like domains 1 |
| 220578_at | 0.007217 | -2.8560819 | ADAMTSL4 | ADAMTS like 4 |
| 207352_s_at | 0.002309 | -2.8486711 | GABRB2 | gamma-aminobutyric acid type A receptor beta2 subunit |
| 210454_s_at | 0.001107 | -2.8474203 | KCNJ6 | potassium voltage-gated channel subfamily J member 6 |
| 207008_at | 0.003971 | 2.8425509 | CXCR2 | C-X-C motif chemokine receptor 2 |
| 213533_at | 0.007907 | 2.8422216 | NSG1 | neuron specific gene family member 1 |
| 204134_at | 0.002599 | -2.8380545 | PDE2A | phosphodiesterase 2A |
| 209540_at | 0.000814 | -2.8350905 | IGF1 | insulin like growth factor 1 |
| 207901_at | 0.004712 | 2.8313264 | IL12B | interleukin 12B |
| 222071_s_at | 0.004988 | 2.8294034 | SLCO4C1 | solute carrier organic anion transporter family member 4C1 |
| 215644_at | 0.003163 | 2.827568 | ZNF518A | zinc finger protein 518A |
| 203695_s_at | 0.000719 | 2.822047 | DFNA5 | DFNA5, deafness associated tumor suppressor |
| 203809_s_at | 0.005628 | 2.8209058 | AKT2 | AKT serine/threonine kinase 2 |
| 204187_at | 0.00631 | 2.8088557 | GMPR | guanosine monophosphate reductase |
| 211171_s_at | 0.000321 | 2.8062084 | PDE10A | phosphodiesterase 10A |
| 211803_at | 0.004648 | 2.8039565 | CDK2 | cyclin dependent kinase 2 |
| 205899_at | 0.001688 | 2.8017812 | CCNA1 | cyclin A1 |
| 206058_at | 0.001282 | -2.7984062 | SLC6A12 | solute carrier family 6 member 12 |
| 206660_at | 0.002003 | 2.7866791 | IGLL1 | immunoglobulin lambda like polypeptide 1 |
| 218621_at | 0.000157 | 2.7866224 | HEMK1 | HemK methyltransferase family member 1 |
| 216298_at | 0.006677 | 2.7836424 | TARP | TCR gamma alternate reading frame protein |
| 217517_x_at | 0.000334 | -2.7644395 | SRPK2 | SRSF protein kinase 2 |
| 220233_at | 0.007333 | 2.7483802 | FBXO17 | F-box protein 17 |
| 207359_at | 0.000673 | 2.7448064 | CAMKK2 | calcium/calmodulin dependent protein kinase kinase 2 |
| 222166_at | 0.001628 | 2.7352673 | C9orf16 | chromosome 9 open reading frame 16 |
| 207296_at | 0.009749 | 2.7351266 | ZNF343 | zinc finger protein 343 |
| 222100_at | 0.003161 | 2.7216362 | CYP2E1 | cytochrome P450 family 2 subfamily E member 1 |
| 207152_at | 0.000607 | 2.7099514 | NTRK2 | neurotrophic receptor tyrosine kinase 2 |
| 216994_s_at | 0.007293 | 2.7062395 | RUNX2 | runt related transcription factor 2 |
| 220077_at | 0.006284 | -2.7013803 | CCDC134 | coiled-coil domain containing 134 |
| 211598_x_at | 0.001656 | 2.668124 | VIPR2 | vasoactive intestinal peptide receptor 2 |
| 209070_s_at | 0.001196 | 2.6624671 | RGS5 | regulator of G-protein signaling 5 |
| 206579_at | 0.001616 | 2.6569029 | ZKSCAN8 | zinc finger with KRAB and SCAN domains 8 |
| 220274_at | 0.001197 | -2.651972 | IQCA1 | IQ motif containing with AAA domain 1 |
| 205568_at | 0.0005 | -2.6413461 | AQP9 | aquaporin 9 |
| 216986_s_at | 0.002053 | -2.6338961 | IRF4 | interferon regulatory factor 4 |
| 214506_at | 0.00042 | 2.6268551 | GPR182 | G protein-coupled receptor 182 |
| 213986_s_at | 0.006124 | 2.6239901 | TMEM259 | transmembrane protein 259 |
| 203382_s_at | 0.00692 | 2.6128447 | APOE | apolipoprotein E |
| 211210_x_at | 0.000228 | 2.6035085 | SH2D1A | SH2 domain containing 1A |
| 207675_x_at | 0.00289 | 2.5967061 | ARTN | artemin |
| 217312_s_at | 0.0047 | 2.581932 | COL7A1 | collagen type VII alpha 1 chain |
| 206616_s_at | 0.00337 | -2.5680315 | ADAM22 | ADAM metallopeptidase domain 22 |
| 207913_at | 0.001824 | 2.5679429 | CYP2F1 | cytochrome P450 family 2 subfamily F member 1 |
| 214008_at | 0.009195 | -2.5633295 | TWF1 | twinfilin actin binding protein 1 |
| 215916_at | 0.005023 | -2.5628724 | CHRNE | cholinergic receptor nicotinic epsilon subunit |
| 214701_s_at | 0.008698 | -2.5626578 | FN1 | fibronectin 1 |
| 216610_at | 0.000265 | 2.5546023 | SLC6A2 | solute carrier family 6 member 2 |
| 216355_at | 0.008324 | 2.5505854 | PCDHB17P | protocadherin beta 17 pseudogene |
| 210702_s_at | 0.008594 | -2.5459712 | PTGIS | prostaglandin I2 (prostacyclin) synthase |
| 203977_at | 0.000462 | 2.5397951 | TAZ | tafazzin |
| 214834_at | 0.006527 | 2.5363641 | PWAR5 | Prader Willi/Angelman region RNA 5 |
| 211772_x_at | 0.006984 | 2.5349484 | CHRNA3 | cholinergic receptor nicotinic alpha 3 subunit |
| 220480_at | 0.000559 | -2.5227484 | HAND2 | heart and neural crest derivatives expressed 2 |
| 210833_at | 0.004771 | 2.5227116 | PTGER3 | prostaglandin E receptor 3 |
| 212940_at | 0.001511 | 2.5224565 | COL6A1 | collagen type VI alpha 1 chain |
| 213925_at | 0.002148 | 2.517895 | STUM | stum, mechanosensory transduction mediator homolog |
| 202340_x_at | 0.004828 | -2.5084762 | NR4A1 | nuclear receptor subfamily 4 group A member 1 |
| 222372_at | 0.009971 | -2.5072677 | MAGI1 | membrane associated guanylate kinase, WW and PDZ domain containing 1 |
| 210383_at | 0.002715 | 2.4797217 | SCN1A | sodium voltage-gated channel alpha subunit 1 |
| 211624_s_at | 0.00234 | 2.4680278 | DRD2 | dopamine receptor D2 |
| 211419_s_at | 0.002868 | 2.4673106 | CHN2 | chimerin 2 |
| 204416_x_at | 0.000739 | 2.4432837 | APOC1 | apolipoprotein C1 |
| 214301_s_at | 0.008755 | 2.4314374 | DPYSL4 | dihydropyrimidinase like 4 |
| 217179_x_at | 0.006281 | 2.4306416 | BMS1P20 | BMS1, ribosome biogenesis factor pseudogene 20 |
| 208111_at | 0.009311 | -2.4181261 | AVPR2 | arginine vasopressin receptor 2 |
| 204996_s_at | 0.005198 | -2.4117561 | CDK5R1 | cyclin dependent kinase 5 regulatory subunit 1 |
| 217553_at | 0.009746 | 2.4083802 | STEAP1B | STEAP family member 1B |
| 210736_x_at | 0.006094 | 2.4050312 | DTNA | dystrobrevin alpha |
| 207376_at | 0.000572 | -2.3762893 | VENTX | VENT homeobox |
| 202652_at | 0.000683 | -2.3760507 | APBB1 | amyloid beta precursor protein binding family B member 1 |
| 210065_s_at | 0.000875 | 2.3738429 | UPK1B | uroplakin 1B |
| 220430_at | 0.003396 | 2.3723507 | FAM110D | family with sequence similarity 110 member D |
| 204693_at | 0.004782 | 2.3663143 | CDC42EP1 | CDC42 effector protein 1 |
| 212935_at | 0.003167 | 2.3631628 | MCF2L | MCF.2 cell line derived transforming sequence like |
| 206368_at | 0.000972 | -2.3621837 | CPLX2 | complexin 2 |
| 206403_at | 0.000848 | 2.3579798 | ZNF536 | zinc finger protein 536 |
| 204482_at | 0.001778 | 2.3506763 | CLDN5 | claudin 5 |
| 216898_s_at | 0.004869 | 2.3494418 | COL4A3 | collagen type IV alpha 3 chain |
| 216919_at | 0.009591 | 2.3339934 | TP53I11 | tumor protein p53 inducible protein 11 |
| 205111_s_at | 0.002075 | -2.3239367 | PLCE1 | phospholipase C epsilon 1 |
| 207666_x_at | 0.003959 | 2.3227854 | SSX3 | SSX family member 3 |
| 219780_at | 0.003462 | 2.3214275 | ZNF771 | zinc finger protein 771 |
| 206001_at | 0.002238 | -2.3152578 | NPY | neuropeptide Y |
| 202018_s_at | 0.008103 | -2.2990074 | LTF | lactotransferrin |
| 205735_s_at | 0.005812 | 2.2961609 | AFF3 | AF4/FMR2 family member 3 |
| 209423_s_at | 0.004173 | 2.28036 | PHF20 | PHD finger protein 20 |
| 207252_at | 0.00492 | -2.2673423 | INE1 | inactivation escape 1 (non-protein coding) |
| 216853_x_at | 0.008187 | 2.266246 | IGLJ3 | immunoglobulin lambda joining 3 |
| 206596_s_at | 0.00894 | -2.2573504 | NRL | neural retina leucine zipper |
| 220294_at | 0.008167 | -2.2481736 | KCNV1 | potassium voltage-gated channel modifier subfamily V member 1 |
| 222022_at | 0.002752 | -2.2420592 | DTX3 | deltex E3 ubiquitin ligase 3 |
| 216327_s_at | 0.007374 | 2.233841 | SIGLEC8 | sialic acid binding Ig like lectin 8 |
| 207067_s_at | 0.005432 | -2.2285786 | HDC | histidine decarboxylase |
| 204122_at | 0.001561 | -2.2233669 | TYROBP | TYRO protein tyrosine kinase binding protein |
| 202902_s_at | 0.002628 | 2.2213088 | CTSS | cathepsin S |
| 211799_x_at | 0.00791 | 2.2144046 | HLA-C | major histocompatibility complex, class I, C |
| 204625_s_at | 0.004342 | 2.2106593 | ITGB3 | integrin subunit beta 3 |
| 207784_at | 0.009312 | 2.2055293 | ARSD | arylsulfatase D |
| 202686_s_at | 0.004068 | -2.1974311 | AXL | AXL receptor tyrosine kinase |
| 214601_at | 0.005742 | 2.1948648 | TPH1 | tryptophan hydroxylase 1 |
| 209888_s_at | 0.008721 | -2.1911813 | MYL1 | myosin light chain 1 |
| 210721_s_at | 0.000906 | -2.1903582 | PAK5 | p21 (RAC1) activated kinase 5 |
| 206360_s_at | 0.000331 | 2.1776095 | SOCS3 | suppressor of cytokine signaling 3 |
| 204582_s_at | 0.003154 | -2.1715665 | KLK3 | kallikrein related peptidase 3 |
| 213715_s_at | 0.009514 | 2.1702614 | KANK3 | KN motif and ankyrin repeat domains 3 |
| 214619_at | 0.005781 | 2.1621295 | CRHR1 | corticotropin releasing hormone receptor 1 |
| 213110_s_at | 0.005878 | 2.1594109 | COL4A5 | collagen type IV alpha 5 chain |
| 205883_at | 0.004035 | 2.1590022 | ZBTB16 | zinc finger and BTB domain containing 16 |
| 214523_at | 0.001104 | 2.1448702 | CEBPE | CCAAT/enhancer binding protein epsilon |
| 221324_at | 0.007916 | -2.1403513 | TAS2R1 | taste 2 receptor member 1 |
| 220680_at | 0.000709 | 2.1357326 | RAVER2 | ribonucleoprotein, PTB binding 2 |
| 208457_at | 0.002142 | 2.1047097 | GABRD | gamma-aminobutyric acid type A receptor delta subunit |
| 216265_x_at | 0.004896 | 2.1030177 | MYH7 | myosin, heavy chain 7, cardiac muscle, beta |
| 203904_x_at | 0.003668 | 2.0995981 | CD82 | CD82 molecule |
| 205515_at | 0.001158 | 2.09365 | PRSS12 | protease, serine 12 |
| 220337_at | 0.005837 | -2.0919741 | NGB | neuroglobin |
| 213350_at | 0.000508 | -2.0860014 | RPS11 | ribosomal protein S11 |
| 220674_at | 0.000568 | -2.0843369 | CD22 | CD22 molecule |
| 203485_at | 0.00505 | 2.0822835 | RTN1 | reticulon 1 |
| 220745_at | 0.005591 | -2.0591823 | IL19 | interleukin 19 |
| 221011_s_at | 0.003383 | 2.0553796 | LBH | limb bud and heart development |
| 216727_at | 0.006371 | 2.0527679 | STK38 | serine/threonine kinase 38 |
| 210366_at | 0.005209 | 2.0523399 | SLCO1B1 | solute carrier organic anion transporter family member 1B1 |
| 214926_at | 0.001599 | -2.0439301 | SPTAN1 | spectrin alpha, non-erythrocytic 1 |
| 206168_at | 0.001293 | 2.0313109 | ZC3H7B | zinc finger CCCH-type containing 7B |
| 214372_x_at | 0.007806 | -2.0267116 | ERN2 | endoplasmic reticulum to nucleus signaling 2 |
| 214520_at | 0.002568 | 2.0211851 | FOXC2 | forkhead box C2 |
| 212992_at | 0.009899 | 2.0043224 | AHNAK2 | AHNAK nucleoprotein 2 |
| 205910_s_at | 0.001664 | -2.0033738 | CEL | carboxyl ester lipase |
| 209763_at | 0.004061 | -2.0024202 | CHRDL1 | chordin like 1 |
| 202627_s_at | 0.001889 | 1.9992255 | SERPINE1 | serpin family E member 1 |
| 216312_at | 0.006057 | -1.982599 | ATP2B3 | ATPase plasma membrane Ca2+ transporting 3 |
| 213958_at | 0.004617 | 1.9751653 | CD6 | CD6 molecule |
| 205186_at | 0.00244 | 1.9578652 | DNALI1 | dynein axonemal light intermediate chain 1 |
| 211214_s_at | 0.006232 | 1.9383749 | DAPK1 | death associated protein kinase 1 |
| 207362_at | 0.007681 | 1.9261598 | SLC30A4 | solute carrier family 30 member 4 |
| 206896_s_at | 0.002078 | 1.9112834 | GNG7 | G protein subunit gamma 7 |
| 217596_at | 0.001023 | 1.9039834 | UPF3A | UPF3 regulator of nonsense transcripts homolog A (yeast) |
| 206568_at | 0.001041 | 1.896095 | TNP1 | transition protein 1 |
| 215654_at | 0.006621 | 1.895144 | BCAT2 | branched chain amino acid transaminase 2 |
| 207762_at | 0.000809 | -1.8944275 | LPAL2 | lipoprotein(a) like 2, pseudogene |
| 211447_s_at | 0.008883 | -1.8857497 | PDE4A | phosphodiesterase 4A |
| 210666_at | 0.000926 | -1.882301 | IDS | iduronate 2-sulfatase |
| 209655_s_at | 0.000443 | 1.878588 | TMEM47 | transmembrane protein 47 |
| 211405_x_at | 0.002256 | 1.8677124 | IFNA17 | interferon alpha 17 |
| 203325_s_at | 0.000534 | 1.8516346 | COL5A1 | collagen type V alpha 1 chain |
| 210629_x_at | 0.002903 | -1.828352 | LST1 | leukocyte specific transcript 1 |
| 221347_at | 0.005924 | 1.8235883 | CHRM5 | cholinergic receptor muscarinic 5 |
| 213167_s_at | 0.006626 | -1.8222029 | SLC5A3 | solute carrier family 5 member 3 |
| 215265_at | 0.000834 | 1.8165538 | EMX1 | empty spiracles homeobox 1 |
| 205413_at | 0.000617 | -1.8148877 | MPPED2 | metallophosphoesterase domain containing 2 |
| 215143_at | 0.005594 | 1.8095476 | DPY19L2P2 | DPY19L2 pseudogene 2 |
| 219726_at | 0.009238 | -1.8037092 | NLGN3 | neuroligin 3 |
| 220053_at | 0.004896 | 1.7948795 | GDF3 | growth differentiation factor 3 |
| 215649_s_at | 0.000763 | 1.7879498 | MVK | mevalonate kinase |
| 220406_at | 0.001177 | -1.7794945 | TGFB2 | transforming growth factor beta 2 |
| 215300_s_at | 0.001016 | 1.7790302 | FMO5 | flavin containing monooxygenase 5 |
| 207594_s_at | 0.003546 | -1.7663318 | SYNJ1 | synaptojanin 1 |
| 211414_at | 0.004676 | -1.763006 | GLS | glutaminase |
| 215065_at | 0.001237 | -1.7492037 | PHF8 | PHD finger protein 8 |
| 207761_s_at | 0.001538 | 1.7317102 | METTL7A | methyltransferase like 7A |
| 220928_s_at | 0.00066 | -1.7302424 | PRDM16 | PR/SET domain 16 |
| 216917_s_at | 0.005036 | 1.7294924 | SYCP1 | synaptonemal complex protein 1 |
| 208167_s_at | 0.007008 | 1.7227399 | MMP16 | matrix metallopeptidase 16 |
| 210420_at | 0.003274 | 1.7216391 | SLC24A1 | solute carrier family 24 member 1 |
| 212258_s_at | 0.008276 | -1.7197326 | SMARCA2 | SWI/SNF related, matrix associated, actin dependent regulator of chromatin, subfamily a, member 2 |
| 205876_at | 0.002574 | 1.7157536 | LIFR | leukemia inhibitory factor receptor alpha |
| 220218_at | 0.009757 | -1.7095872 | SPATA6L | spermatogenesis associated 6 like |
| 213955_at | 0.000737 | -1.7071501 | MYOZ3 | myozenin 3 |
| 205121_at | 0.003794 | 1.7015294 | SGCB | sarcoglycan beta |
| 221901_at | 0.001616 | -1.6951461 | KIAA1644 | KIAA1644 |
| 215735_s_at | 0.001572 | 1.6896808 | TSC2 | tuberous sclerosis 2 |
| 206817_x_at | 0.003072 | 1.6873461 | CELF3 | CUGBP, Elav-like family member 3 |
| 210604_at | 0.000987 | 1.6826743 | GNAT2 | G protein subunit alpha transducin 2 |
| 211559_s_at | 0.00137 | 1.680613 | CCNG2 | cyclin G2 |
| 215977_x_at | 0.002367 | -1.6789931 | GK | glycerol kinase |
| 217341_at | 0.006323 | 1.678332 | DNM1 | dynamin 1 |
| 208077_at | 0.003187 | -1.6615828 | C9orf38 | chromosome 9 open reading frame 38 |
| 222336_at | 0.009791 | -1.6460219 | SMIM14 | small integral membrane protein 14 |
| 220380_at | 0.001326 | -1.642345 | DNASE2B | deoxyribonuclease 2 beta |
| 207348_s_at | 0.001149 | 1.6421754 | LIG3 | DNA ligase 3 |
| 215652_at | 0.005371 | -1.6287213 | SDHD | succinate dehydrogenase complex subunit D |
| 217231_s_at | 0.003494 | -1.6228934 | MAST1 | microtubule associated serine/threonine kinase 1 |
| 209839_at | 0.003225 | 1.6140458 | DNM3 | dynamin 3 |
| 206820_at | 0.008634 | 1.6080814 | AGFG2 | ArfGAP with FG repeats 2 |
| 200907_s_at | 0.001358 | 1.6056971 | PALLD | palladin, cytoskeletal associated protein |
| 203710_at | 0.003617 | 1.5981414 | ITPR1 | inositol 1,4,5-trisphosphate receptor type 1 |
| 221606_s_at | 0.002677 | 1.5956489 | HMGN5 | high mobility group nucleosome binding domain 5 |
| 219113_x_at | 0.007075 | -1.5941018 | HSD17B14 | hydroxysteroid 17-beta dehydrogenase 14 |
| 219475_at | 0.007023 | -1.5918067 | OSGIN1 | oxidative stress induced growth inhibitor 1 |
| 220411_x_at | 0.008925 | -1.5817261 | PODNL1 | podocan like 1 |
| 219956_at | 0.008692 | 1.5671524 | GALNT6 | polypeptide N-acetylgalactosaminyltransferase 6 |
| 214385_s_at | 0.002827 | -1.5606995 | MUC5AC | mucin 5AC, oligomeric mucus/gel-forming |
| 207592_s_at | 0.009003 | 1.5558728 | HCN2 | hyperpolarization activated cyclic nucleotide gated potassium channel 2 |
| 205348_s_at | 0.008176 | -1.5480641 | DYNC1I1 | dynein cytoplasmic 1 intermediate chain 1 |
| 220603_s_at | 0.002201 | -1.5325145 | MCTP2 | multiple C2 and transmembrane domain containing 2 |
| 203757_s_at | 0.002061 | -1.5303843 | CEACAM6 | carcinoembryonic antigen related cell adhesion molecule 6 |
| 214821_at | 0.004923 | -1.5218986 | SLC25A4 | solute carrier family 25 member 4 |
| 213563_s_at | 0.004737 | -1.5216963 | TUBGCP2 | tubulin gamma complex associated protein 2 |
| 214475_x_at | 0.001852 | -1.5153972 | CAPN3 | calpain 3 |
| 220510_at | 0.002355 | 1.5143575 | RHBG | Rh family B glycoprotein (gene/pseudogene) |
| 220412_x_at | 0.007096 | -1.5128523 | KCNK7 | potassium two pore domain channel subfamily K member 7 |
| 207827_x_at | 0.006527 | 1.5096822 | SNCA | synuclein alpha |
| 211735_x_at | 0.001675 | -1.4993572 | SFTPC | surfactant protein C |
| 220075_s_at | 0.006396 | -1.4936395 | CDHR5 | cadherin related family member 5 |
| 217083_at | 0.00722 | -1.482365 | IGHV3-75 | immunoglobulin heavy variable 3-75 (pseudogene) |
| 204433_s_at | 0.003639 | -1.4659147 | SPATA2 | spermatogenesis associated 2 |
| 201884_at | 0.001125 | -1.4616271 | CEACAM5 | carcinoembryonic antigen related cell adhesion molecule 5 |
| 207924_x_at | 0.004083 | -1.4610546 | PAX8 | paired box 8 |
| 207373_at | 0.003857 | -1.4584115 | HOXD10 | homeobox D10 |
| 214182_at | 0.004954 | -1.4558429 | ARF6 | ADP ribosylation factor 6 |
| 202694_at | 0.002233 | 1.455099 | STK17A | serine/threonine kinase 17a |
| 219223_at | 0.004317 | -1.447712 | CACFD1 | calcium channel flower domain containing 1 |
| 202431_s_at | 0.001255 | 1.4473244 | MYC | v-myc avian myelocytomatosis viral oncogene homolog |
| 205003_at | 0.006206 | 1.4410289 | DOCK4 | dedicator of cytokinesis 4 |
| 215913_s_at | 0.001837 | 1.4374308 | GULP1 | GULP, engulfment adaptor PTB domain containing 1 |
| 206496_at | 0.006765 | -1.4272459 | FMO3 | flavin containing monooxygenase 3 |
| 201859_at | 0.004283 | 1.4158602 | SRGN | serglycin |
| 220584_at | 0.009225 | 1.415292 | PRR36 | proline rich 36 |
| 203736_s_at | 0.004564 | 1.4018362 | PPFIBP1 | PPFIA binding protein 1 |
| 215402_at | 0.008082 | -1.4016736 | APPBP2 | amyloid beta precursor protein binding protein 2 |
| 216793_x_at | 0.005433 | -1.3991033 | LOC100289518 | immunoglobin superfamily member 21 pseudogene |
| 210567_s_at | 0.009872 | 1.3960683 | SKP2 | S-phase kinase-associated protein 2, E3 ubiquitin protein ligase |
| 206531_at | 0.001198 | -1.3951585 | DPF1 | double PHD fingers 1 |
| 215269_at | 0.002105 | -1.394774 | TRAPPC10 | trafficking protein particle complex 10 |
| 213354_s_at | 0.002328 | -1.3925942 | NR2F6 | nuclear receptor subfamily 2 group F member 6 |
| 202944_at | 0.00133 | 1.3863984 | NAGA | alpha-N-acetylgalactosaminidase |
| 209292_at | 0.004042 | -1.383243 | ID4 | inhibitor of DNA binding 4, HLH protein |
| 207745_at | 0.002407 | 1.3814057 | CABP2 | calcium binding protein 2 |
| 206355_at | 0.008809 | 1.3797794 | GNAL | G protein subunit alpha L |
| 210040_at | 0.006401 | 1.3676298 | SLC12A5 | solute carrier family 12 member 5 |
| 203949_at | 0.003263 | -1.3565633 | MPO | myeloperoxidase |
| 205673_s_at | 0.001453 | 1.354133 | ASB9 | ankyrin repeat and SOCS box containing 9 |
| 207601_at | 0.005248 | 1.3502231 | SULT1B1 | sulfotransferase family 1B member 1 |
| 203391_at | 0.001981 | -1.3481339 | FKBP2 | FK506 binding protein 2 |
| 211332_x_at | 0.002776 | -1.3428506 | HFE | hemochromatosis |
| 207777_s_at | 0.009963 | 1.334045 | SP140 | SP140 nuclear body protein |
| 202648_at | 0.007082 | -1.3199372 | TCF3 | transcription factor 3 |
| 210837_s_at | 0.003605 | -1.3089989 | PDE4D | phosphodiesterase 4D |
| 204165_at | 0.001534 | 1.3058265 | WASF1 | WAS protein family member 1 |
| 219153_s_at | 0.004605 | -1.3041268 | THSD4 | thrombospondin type 1 domain containing 4 |
| 211259_s_at | 0.002081 | 1.3036879 | BMP7 | bone morphogenetic protein 7 |
| 201468_s_at | 0.002627 | -1.3023946 | NQO1 | NAD(P)H quinone dehydrogenase 1 |
| 204636_at | 0.005329 | -1.3023795 | COL17A1 | collagen type XVII alpha 1 chain |
| 204428_s_at | 0.008243 | -1.3012768 | LCAT | lecithin-cholesterol acyltransferase |
| 210640_s_at | 0.003223 | 1.2972976 | GPER1 | G protein-coupled estrogen receptor 1 |
| 220444_at | 0.002726 | -1.2930078 | ZNF557 | zinc finger protein 557 |
| 200982_s_at | 0.001685 | 1.2911508 | ANXA6 | annexin A6 |
| 214295_at | 0.007833 | 1.2783814 | KIAA0485 | uncharacterized LOC57235 |
| 209114_at | 0.00145 | -1.2716585 | TSPAN1 | tetraspanin 1 |
| 218999_at | 0.003178 | 1.2620467 | TMEM140 | transmembrane protein 140 |
| 210321_at | 0.009986 | 1.2604517 | GZMH | granzyme H |
| 205898_at | 0.001596 | 1.2580024 | CX3CR1 | C-X3-C motif chemokine receptor 1 |
| 219313_at | 0.008601 | 1.2560961 | GRAMD1C | GRAM domain containing 1C |
| 209687_at | 0.004151 | -1.2477037 | CXCL12 | C-X-C motif chemokine ligand 12 |
| 214682_at | 0.009437 | -1.2472827 | PKD1P1 | polycystin 1, transient receptor potential channel interacting pseudogene 1 |
| 220124_at | 0.008108 | -1.2420942 | GAN | gigaxonin |
| 208319_s_at | 0.002153 | 1.2312739 | RBM3 | RNA binding motif (RNP1, RRM) protein 3 |
| 217437_s_at | 0.007056 | 1.2287157 | TACC1 | transforming acidic coiled-coil containing protein 1 |
| 222008_at | 0.006769 | -1.2265778 | COL9A1 | collagen type IX alpha 1 chain |
| 207484_s_at | 0.00822 | 1.2190586 | EHMT2 | euchromatic histone lysine methyltransferase 2 |
| 204472_at | 0.006391 | 1.2020621 | GEM | GTP binding protein overexpressed in skeletal muscle |
| 205064_at | 0.004221 | -1.2007933 | SPRR1B | small proline rich protein 1B |
| 218750_at | 0.001916 | -1.200687 | TAF1D | TATA-box binding protein associated factor, RNA polymerase I subunit D |
| 217996_at | 0.002234 | -1.1971689 | PHLDA1 | pleckstrin homology like domain family A member 1 |
| 220176_at | 0.004193 | 1.190004 | NUBPL | nucleotide binding protein like |
| 203685_at | 0.006294 | 1.1859707 | BCL2 | BCL2, apoptosis regulator |
| 203795_s_at | 0.002205 | -1.1853602 | BCL7A | BCL tumor suppressor 7A |
| 218151_x_at | 0.008221 | -1.177724 | SLC52A2 | solute carrier family 52 member 2 |
| 208366_at | 0.006659 | -1.1763319 | PCDH11X | protocadherin 11 X-linked |
| 201311_s_at | 0.006854 | 1.1725244 | SH3BGRL | SH3 domain binding glutamate rich protein like |
| 213791_at | 0.005022 | -1.172175 | PENK | proenkephalin |
| 213186_at | 0.007538 | 1.1721142 | DZIP3 | DAZ interacting zinc finger protein 3 |
| 207037_at | 0.004504 | 1.1699078 | TNFRSF11A | TNF receptor superfamily member 11a |
| 203570_at | 0.001847 | -1.1690546 | LOXL1 | lysyl oxidase like 1 |
| 205694_at | 0.003347 | 1.168075 | TYRP1 | tyrosinase related protein 1 |
| 208448_x_at | 0.002386 | 1.1666855 | IFNA16 | interferon alpha 16 |
| 203958_s_at | 0.002704 | 1.164459 | ZBTB40 | zinc finger and BTB domain containing 40 |
| 208078_s_at | 0.00228 | 1.1610601 | SIK1 | salt inducible kinase 1 |
| 216120_s_at | 0.002692 | 1.1570836 | ATP2B2 | ATPase plasma membrane Ca2+ transporting 2 |
| 213174_at | 0.008657 | -1.1541211 | TTC9 | tetratricopeptide repeat domain 9 |
| 204311_at | 0.002006 | 1.153273 | ATP1B2 | ATPase Na+/K+ transporting subunit beta 2 |
| 32625_at | 0.003788 | -1.1521821 | NPR1 | natriuretic peptide receptor 1 |
| 221197_s_at | 0.004351 | -1.1513147 | CHAT | choline O-acetyltransferase |
| 220290_at | 0.008971 | -1.1420224 | AIM1L | absent in melanoma 1-like |
| 203439_s_at | 0.002084 | -1.1407536 | STC2 | stanniocalcin 2 |
| 209844_at | 0.006345 | -1.1407146 | HOXB13 | homeobox B13 |
| 218031_s_at | 0.002864 | 1.137874 | FOXN3 | forkhead box N3 |
| 219106_s_at | 0.008911 | -1.1371553 | KLHL41 | kelch like family member 41 |
| 219028_at | 0.002942 | -1.1367258 | HIPK2 | homeodomain interacting protein kinase 2 |
| 212909_at | 0.002174 | 1.1347984 | LYPD1 | LY6/PLAUR domain containing 1 |
| 219279_at | 0.008191 | 1.1300672 | DOCK10 | dedicator of cytokinesis 10 |
| 210036_s_at | 0.006914 | 1.127879 | KCNH2 | potassium voltage-gated channel subfamily H member 2 |
| 221729_at | 0.002257 | 1.1274484 | COL5A2 | collagen type V alpha 2 chain |
| 220005_at | 0.003716 | -1.1266213 | P2RY13 | purinergic receptor P2Y13 |
| 213089_at | 0.006908 | -1.1241935 | LOC100272216 | uncharacterized LOC100272216 |
| 200973_s_at | 0.003502 | 1.1205362 | TSPAN3 | tetraspanin 3 |
| 210855_at | 0.003386 | -1.1181637 | GREB1 | growth regulation by estrogen in breast cancer 1 |
| 207142_at | 0.008231 | -1.1164583 | KCNJ3 | potassium voltage-gated channel subfamily J member 3 |
| 219287_at | 0.003682 | 1.1155512 | KCNMB4 | potassium calcium-activated channel subfamily M regulatory beta subunit 4 |
| 210830_s_at | 0.007229 | 1.1142618 | PON2 | paraoxonase 2 |
| 212969_x_at | 0.003894 | 1.1128116 | EML3 | echinoderm microtubule associated protein like 3 |
| 222152_at | 0.003602 | -1.1064286 | PDCD6 | programmed cell death 6 |
| 220477_s_at | 0.009502 | 1.1029197 | TMEM230 | transmembrane protein 230 |
| 206696_at | 0.004842 | 1.1023227 | GPR143 | G protein-coupled receptor 143 |
| 201170_s_at | 0.003161 | 1.0997738 | BHLHE40 | basic helix-loop-helix family member e40 |
| 205549_at | 0.004952 | -1.0985322 | PCP4 | Purkinje cell protein 4 |
| 204044_at | 0.004843 | 1.0966809 | QPRT | quinolinate phosphoribosyltransferase |
| 207826_s_at | 0.004105 | -1.0930266 | ID3 | inhibitor of DNA binding 3, HLH protein |
| 213736_at | 0.00698 | -1.092379 | COX5B | cytochrome c oxidase subunit 5B |
| 217875_s_at | 0.006673 | 1.0869521 | PMEPA1 | prostate transmembrane protein, androgen induced 1 |
| 208388_at | 0.006263 | -1.084914 | NR2E3 | nuclear receptor subfamily 2 group E member 3 |
| 213478_at | 0.003221 | -1.0840929 | KAZN | kazrin, periplakin interacting protein |
| 214266_s_at | 0.006164 | 1.0832035 | PDLIM7 | PDZ and LIM domain 7 |
| 216259_at | 0.004872 | -1.0808527 | LOC100506699 | uncharacterized LOC100506699 |
| 220390_at | 0.004989 | 1.0791608 | AGBL2 | ATP/GTP binding protein like 2 |
| 201110_s_at | 0.003707 | 1.0773549 | THBS1 | thrombospondin 1 |
| 212806_at | 0.005845 | 1.0675603 | PRUNE2 | prune homolog 2 |
| 219074_at | 0.003914 | -1.0673373 | TMEM184C | transmembrane protein 184C |
| 209988_s_at | 0.005474 | 1.0612905 | ASCL1 | achaete-scute family bHLH transcription factor 1 |
| 213789_at | 0.00975 | -1.0520464 | EBP | emopamil binding protein (sterol isomerase) |
| 210560_at | 0.004128 | 1.0509968 | GBX2 | gastrulation brain homeobox 2 |
| 214958_s_at | 0.005363 | 1.0501342 | TMC6 | transmembrane channel like 6 |
| 204695_at | 0.00291 | -1.044209 | CDC25A | cell division cycle 25A |
| 203942_s_at | 0.005961 | 1.0426185 | MARK2 | microtubule affinity regulating kinase 2 |
| 220720_x_at | 0.002798 | -1.0401129 | MZT2B | mitotic spindle organizing protein 2B |
| 205226_at | 0.006165 | 1.0374378 | PDGFRL | platelet derived growth factor receptor like |
| 204589_at | 0.006016 | 1.0371151 | NUAK1 | NUAK family kinase 1 |
| 213548_s_at | 0.006279 | 1.0366423 | CDV3 | CDV3 homolog |
| 203032_s_at | 0.005422 | 1.0306299 | FH | fumarate hydratase |
| 208931_s_at | 0.007253 | 1.0290551 | ILF3 | interleukin enhancer binding factor 3 |
| 214395_x_at | 0.005485 | -1.0269412 | EEF1D | eukaryotic translation elongation factor 1 delta |
| 207465_at | 0.007778 | 1.024357 | LOC100127886 | uncharacterized LOC100127886 |
| 222196_at | 0.009152 | 1.0159507 | LOC389906 | zinc finger protein 839 pseudogene |
| 217197_x_at | 0.004308 | 1.0149095 | N4BP2L1 | NEDD4 binding protein 2 like 1 |
| 202619_s_at | 0.005665 | 1.0113707 | PLOD2 | procollagen-lysine,2-oxoglutarate 5-dioxygenase 2 |
| 207038_at | 0.008461 | 1.010339 | SLC16A6 | solute carrier family 16 member 6 |
| 218569_s_at | 0.004463 | 1.0097566 | KBTBD4 | kelch repeat and BTB domain containing 4 |
| 204698_at | 0.008485 | -1.0097412 | ISG20 | interferon stimulated exonuclease gene 20 |
| 219985_at | 0.009811 | -1.0055009 | HS3ST3A1 | heparan sulfate-glucosamine 3-sulfotransferase 3A1 |
