# Supplementary material for: Potential Targets and Action Mechanism of Gastrodin in the Treatment of Attention-Deficit/Hyperactivity Disorder: Bioinformatics and Network Pharmacology Analysis
Source: Evid Based Complement Alternat Med. 2022 Sep 12;2022:3607053. doi: 10.1155/2022/3607053 (PMC9484880; doi:10.1155/2022/3607053)
Supplement: Supplementary Materials — Supplementary Table 1: the 460 DEGs in GSE85871. Supplementary Table 2: the known targets of gastrodin in four drug databases. Supplementary Table 3: 584 gastrodin-related drug targets. Supplementary Table 4: the ADHD-related disease genes. [file 3607053.f1.zip › 3607053.f1/Supplementary Table 2.docx]

| ETCM | PubChem | STITCH | SwissTargetPrediction |
| --- | --- | --- | --- |
| LCTL | HMOX1 | NOS1 | CDA |
| LGALS2 | IL6 | NOS2 | TYR |
| LGALS3 | NFE2L2 | IL1B | ADORA2A |
| LGALS7 | PTGS2 | NOS3 | SLC5A2 |
| MB | SOD1 | POR | TDP1 |
| MBL2 | STK11 | MTRR | ADORA1 |
| NCAN | TNF |  | AKR1B1 |
| NUDT9 |  |  | SLC5A1 |
| PTGS1 |  |  | MGAM |
| PYGL |  |  | SLC5A4 |
| PYGM |  |  | FUCA1 |
| SFTPD |  |  | FOLH1 |
| SIGLEC1 |  |  | TREH |
| SMARCA5 |  |  | CA14 |
| TM0024 |  |  | ADA |
| TYR |  |  | ADORA3 |
|  |  |  | SI |
|  |  |  | HK2 |
|  |  |  | HK1 |
|  |  |  | AMY2A |
|  |  |  | IDO1 |
|  |  |  | DTYMK |
|  |  |  | ADK |
|  |  |  | AKR1C3 |
|  |  |  | HPRT1 |
|  |  |  | PTPN1 |
|  |  |  | SRD5A1 |
|  |  |  | EPHX2 |
|  |  |  | NAALAD2 |
|  |  |  | HSPA8 |
|  |  |  | GAPDH |
|  |  |  | GRK1 |
|  |  |  | PNP |
|  |  |  | PTPN11 |
|  |  |  | IGFBP3 |
|  |  |  | CA1 |
|  |  |  | CA12 |
|  |  |  | CA9 |
|  |  |  | HSPA5 |
|  |  |  | AKR1C2 |
|  |  |  | OGA |
|  |  |  | SLC29A1 |
|  |  |  | PIK3CG |
|  |  |  | PIK3CA |
|  |  |  | CYP19A1 |
|  |  |  | MAPK1 |
|  |  |  | MME |
|  |  |  | CDK2 |
|  |  |  | DPP4 |
|  |  |  | CA5A |
|  |  |  | GRIK1 |
|  |  |  | CA6 |
|  |  |  | GRIK2 |
|  |  |  | GRIK3 |
|  |  |  | AHCY |
|  |  |  | FBP1 |
|  |  |  | IGF1R |
|  |  |  | INSR |
|  |  |  | SELL |
|  |  |  | SELE |
|  |  |  | SELP |
|  |  |  | CA3 |
|  |  |  | CASP3 |
|  |  |  | PYGL |
|  |  |  | PYGM |
|  |  |  | CA2 |
|  |  |  | GGH |
|  |  |  | ENPEP |
|  |  |  | PTGS1 |
|  |  |  | GSK3B |
|  |  |  | P2RX3 |
|  |  |  | CDK1 |
|  |  |  | EDNRA |
|  |  |  | MMP13 |
|  |  |  | MMP1 |
|  |  |  | MMP7 |
|  |  |  | MMP8 |
|  |  |  | CASP6 |
|  |  |  | CASP7 |
|  |  |  | CASP8 |
|  |  |  | CFTR |
|  |  |  | GNPAT |
|  |  |  | CASP1 |
|  |  |  | CASP2 |
|  |  |  | ECE1 |
|  |  |  | ERN1 |
|  |  |  | MMP16 |
|  |  |  | FTO |
|  |  |  | MMP9 |
|  |  |  | MMP14 |
|  |  |  | DRD2 |
|  |  |  | DRD4 |
|  |  |  | GPR35 |
|  |  |  | ADORA2B |
|  |  |  | PLA2G4B |
|  |  |  | NGFR |
|  |  |  | AKR1C1 |
|  |  |  | KDM4D |
|  |  |  | KDM4C |
|  |  |  | HRAS |
|  |  |  | CCNA1 |
|  |  |  | CCNA2 |
|  |  |  | CCNB1 |
