# Supplementary material for: Potential Targets and Action Mechanism of Gastrodin in the Treatment of Attention-Deficit/Hyperactivity Disorder: Bioinformatics and Network Pharmacology Analysis
Source: Evid Based Complement Alternat Med. 2022 Sep 12;2022:3607053. doi: 10.1155/2022/3607053 (PMC9484880; doi:10.1155/2022/3607053)
Supplement: Supplementary Materials — Supplementary Table 1: the 460 DEGs in GSE85871. Supplementary Table 2: the known targets of gastrodin in four drug databases. Supplementary Table 3: 584 gastrodin-related drug targets. Supplementary Table 4: the ADHD-related disease genes. [file 3607053.f1.zip › 3607053.f1/Supplementary Table 3.docx]

| LCTL |
| --- |
| LGALS2 |
| LGALS3 |
| LGALS7 |
| MB |
| MBL2 |
| NCAN |
| NUDT9 |
| PTGS1 |
| PYGL |
| PYGM |
| SFTPD |
| SIGLEC1 |
| SMARCA5 |
| TM0024 |
| TYR |
| HMOX1 |
| IL6 |
| NFE2L2 |
| PTGS2 |
| SOD1 |
| STK11 |
| TNF |
| CDA |
| ADORA2A |
| SLC5A2 |
| TDP1 |
| ADORA1 |
| AKR1B1 |
| SLC5A1 |
| MGAM |
| SLC5A4 |
| FUCA1 |
| FOLH1 |
| TREH |
| CA14 |
| ADA |
| ADORA3 |
| SI |
| HK2 |
| HK1 |
| AMY2A |
| IDO1 |
| DTYMK |
| ADK |
| AKR1C3 |
| HPRT1 |
| PTPN1 |
| SRD5A1 |
| EPHX2 |
| NAALAD2 |
| HSPA8 |
| GAPDH |
| GRK1 |
| PNP |
| PTPN11 |
| IGFBP3 |
| CA1 |
| CA12 |
| CA9 |
| HSPA5 |
| AKR1C2 |
| OGA |
| SLC29A1 |
| PIK3CG |
| PIK3CA |
| CYP19A1 |
| MAPK1 |
| MME |
| CDK2 |
| DPP4 |
| CA5A |
| GRIK1 |
| CA6 |
| GRIK2 |
| GRIK3 |
| AHCY |
| FBP1 |
| IGF1R |
| INSR |
| SELL |
| SELE |
| SELP |
| CA3 |
| CASP3 |
| CA2 |
| GGH |
| ENPEP |
| GSK3B |
| P2RX3 |
| CDK1 |
| EDNRA |
| MMP13 |
| MMP1 |
| MMP7 |
| MMP8 |
| CASP6 |
| CASP7 |
| CASP8 |
| CFTR |
| GNPAT |
| CASP1 |
| CASP2 |
| ECE1 |
| ERN1 |
| MMP16 |
| FTO |
| MMP9 |
| MMP14 |
| DRD2 |
| DRD4 |
| GPR35 |
| ADORA2B |
| PLA2G4B |
| NGFR |
| AKR1C1 |
| KDM4D |
| KDM4C |
| HRAS |
| CCNA1 |
| CCNA2 |
| CCNB1 |
| NOS1 |
| NOS2 |
| IL1B |
| NOS3 |
| POR |
| MTRR |
| TEK |
| AFF2 |
| MBNL2 |
| CHI3L1 |
| PRTN3 |
| CNTLN |
| OCLM |
| IFNA1 |
| CYP1A1 |
| AREG |
| TRDV3 |
| CXCL11 |
| JRK |
| PLEK |
| KRTAP9-9 |
| SPATA1 |
| NRIP2 |
| SCN2A |
| ABCB11 |
| EDDM3B |
| LAMA2 |
| ZBBX |
| GPX7 |
| CADM4 |
| ZNF749 |
| IVD |
| CPNE7 |
| NPY2R |
| KRTAP1-3 |
| PVRIG |
| CACNA1I |
| ETV1 |
| COL14A1 |
| CALD1 |
| TNFSF8 |
| JUND |
| GIF |
| RNASE1 |
| OR7E2P |
| RANGAP1 |
| NPTX2 |
| PF4V1 |
| CAV3 |
| ANKRD7 |
| MT4 |
| PTGFR |
| MS4A12 |
| NYNRIN |
| SPTBN2 |
| GBP1 |
| PTPN22 |
| NEFL |
| LAMP5 |
| TRAK1 |
| INVS |
| AVIL |
| TGM4 |
| IFNW1 |
| CHRNA10 |
| RIT2 |
| SAA3P |
| PDE7B |
| TRPC3 |
| NEFH |
| IFNA5 |
| AZU1 |
| SPC24 |
| SCN3A |
| TXLNGY |
| KMT2D |
| MINK1 |
| RNASE4 |
| DLGAP2 |
| EFCAB6 |
| BIN1 |
| OR3A3 |
| SLFN12 |
| CLCN7 |
| GH1 |
| TP53AIP1 |
| MBP |
| PLEKHA4 |
| PROC |
| MAGEA4 |
| KCND3 |
| PPFIA2 |
| CNR1 |
| EDA |
| EYA4 |
| TM4SF5 |
| MAF |
| NKX6-1 |
| RFPL1S |
| SCGB2A1 |
| PECAM1 |
| LOC101926913 |
| NPAS1 |
| TDO2 |
| PIWIL1 |
| SNTB1 |
| CLC |
| PVR |
| IGF2BP3 |
| ITGB2 |
| KLRD1 |
| HGF |
| CLTCL1 |
| C1QA |
| TNPO2 |
| KLK2 |
| BLK |
| UBE4B |
| SLC2A1 |
| APC2 |
| SAGE1 |
| TCL6 |
| LCP2 |
| MST1 |
| IRF5 |
| HEG1 |
| ADAMTSL4 |
| GABRB2 |
| KCNJ6 |
| CXCR2 |
| NSG1 |
| PDE2A |
| IGF1 |
| IL12B |
| SLCO4C1 |
| ZNF518A |
| DFNA5 |
| AKT2 |
| GMPR |
| PDE10A |
| SLC6A12 |
| IGLL1 |
| HEMK1 |
| TARP |
| SRPK2 |
| FBXO17 |
| CAMKK2 |
| C9orf16 |
| ZNF343 |
| CYP2E1 |
| NTRK2 |
| RUNX2 |
| CCDC134 |
| VIPR2 |
| RGS5 |
| ZKSCAN8 |
| IQCA1 |
| AQP9 |
| IRF4 |
| GPR182 |
| TMEM259 |
| APOE |
| SH2D1A |
| ARTN |
| COL7A1 |
| ADAM22 |
| CYP2F1 |
| TWF1 |
| CHRNE |
| FN1 |
| SLC6A2 |
| PCDHB17P |
| PTGIS |
| TAZ |
| PWAR5 |
| CHRNA3 |
| HAND2 |
| PTGER3 |
| COL6A1 |
| STUM |
| NR4A1 |
| MAGI1 |
| SCN1A |
| CHN2 |
| APOC1 |
| DPYSL4 |
| BMS1P20 |
| AVPR2 |
| CDK5R1 |
| STEAP1B |
| DTNA |
| VENTX |
| APBB1 |
| UPK1B |
| FAM110D |
| CDC42EP1 |
| MCF2L |
| CPLX2 |
| ZNF536 |
| CLDN5 |
| COL4A3 |
| TP53I11 |
| PLCE1 |
| SSX3 |
| ZNF771 |
| NPY |
| LTF |
| AFF3 |
| PHF20 |
| INE1 |
| IGLJ3 |
| NRL |
| KCNV1 |
| DTX3 |
| SIGLEC8 |
| HDC |
| TYROBP |
| CTSS |
| HLA-C |
| ITGB3 |
| ARSD |
| AXL |
| TPH1 |
| MYL1 |
| PAK5 |
| SOCS3 |
| KLK3 |
| KANK3 |
| CRHR1 |
| COL4A5 |
| ZBTB16 |
| CEBPE |
| TAS2R1 |
| RAVER2 |
| GABRD |
| MYH7 |
| CD82 |
| PRSS12 |
| NGB |
| RPS11 |
| CD22 |
| RTN1 |
| IL19 |
| LBH |
| STK38 |
| SLCO1B1 |
| SPTAN1 |
| ZC3H7B |
| ERN2 |
| FOXC2 |
| AHNAK2 |
| CEL |
| CHRDL1 |
| SERPINE1 |
| ATP2B3 |
| CD6 |
| DNALI1 |
| DAPK1 |
| SLC30A4 |
| GNG7 |
| UPF3A |
| TNP1 |
| BCAT2 |
| LPAL2 |
| PDE4A |
| IDS |
| TMEM47 |
| IFNA17 |
| COL5A1 |
| LST1 |
| CHRM5 |
| SLC5A3 |
| EMX1 |
| MPPED2 |
| DPY19L2P2 |
| NLGN3 |
| GDF3 |
| MVK |
| TGFB2 |
| FMO5 |
| SYNJ1 |
| GLS |
| PHF8 |
| METTL7A |
| PRDM16 |
| SYCP1 |
| SLC24A1 |
| SMARCA2 |
| LIFR |
| SPATA6L |
| MYOZ3 |
| SGCB |
| KIAA1644 |
| TSC2 |
| CELF3 |
| GNAT2 |
| CCNG2 |
| GK |
| DNM1 |
| C9orf38 |
| SMIM14 |
| DNASE2B |
| LIG3 |
| SDHD |
| MAST1 |
| DNM3 |
| AGFG2 |
| PALLD |
| ITPR1 |
| HMGN5 |
| HSD17B14 |
| OSGIN1 |
| PODNL1 |
| GALNT6 |
| MUC5AC |
| HCN2 |
| DYNC1I1 |
| MCTP2 |
| CEACAM6 |
| SLC25A4 |
| TUBGCP2 |
| CAPN3 |
| RHBG |
| KCNK7 |
| SNCA |
| SFTPC |
| CDHR5 |
| IGHV3-75 |
| SPATA2 |
| CEACAM5 |
| PAX8 |
| HOXD10 |
| ARF6 |
| STK17A |
| CACFD1 |
| MYC |
| DOCK4 |
| GULP1 |
| FMO3 |
| SRGN |
| PRR36 |
| PPFIBP1 |
| APPBP2 |
| LOC100289518 |
| SKP2 |
| DPF1 |
| TRAPPC10 |
| NR2F6 |
| NAGA |
| ID4 |
| CABP2 |
| GNAL |
| SLC12A5 |
| MPO |
| ASB9 |
| SULT1B1 |
| FKBP2 |
| HFE |
| SP140 |
| TCF3 |
| PDE4D |
| WASF1 |
| THSD4 |
| BMP7 |
| NQO1 |
| COL17A1 |
| LCAT |
| GPER1 |
| ZNF557 |
| ANXA6 |
| KIAA0485 |
| TSPAN1 |
| TMEM140 |
| GZMH |
| CX3CR1 |
| GRAMD1C |
| CXCL12 |
| PKD1P1 |
| GAN |
| RBM3 |
| TACC1 |
| COL9A1 |
| EHMT2 |
| GEM |
| SPRR1B |
| TAF1D |
| PHLDA1 |
| NUBPL |
| BCL2 |
| BCL7A |
| SLC52A2 |
| PCDH11X |
| SH3BGRL |
| PENK |
| DZIP3 |
| TNFRSF11A |
| LOXL1 |
| TYRP1 |
| IFNA16 |
| ZBTB40 |
| SIK1 |
| ATP2B2 |
| TTC9 |
| ATP1B2 |
| NPR1 |
| CHAT |
| AIM1L |
| STC2 |
| HOXB13 |
| FOXN3 |
| KLHL41 |
| HIPK2 |
| LYPD1 |
| DOCK10 |
| KCNH2 |
| COL5A2 |
| P2RY13 |
| LOC100272216 |
| TSPAN3 |
| GREB1 |
| KCNJ3 |
| KCNMB4 |
| PON2 |
| EML3 |
| PDCD6 |
| TMEM230 |
| GPR143 |
| BHLHE40 |
| PCP4 |
| QPRT |
| ID3 |
| COX5B |
| PMEPA1 |
| NR2E3 |
| KAZN |
| PDLIM7 |
| LOC100506699 |
| AGBL2 |
| THBS1 |
| PRUNE2 |
| TMEM184C |
| ASCL1 |
| EBP |
| GBX2 |
| TMC6 |
| CDC25A |
| MARK2 |
| MZT2B |
| PDGFRL |
| NUAK1 |
| CDV3 |
| FH |
| ILF3 |
| EEF1D |
| LOC100127886 |
| LOC389906 |
| N4BP2L1 |
| PLOD2 |
| SLC16A6 |
| KBTBD4 |
| ISG20 |
| HS3ST3A1 |
