# Supplementary material for: Potential Targets and Action Mechanism of Gastrodin in the Treatment of Attention-Deficit/Hyperactivity Disorder: Bioinformatics and Network Pharmacology Analysis
Source: Evid Based Complement Alternat Med. 2022 Sep 12;2022:3607053. doi: 10.1155/2022/3607053 (PMC9484880; doi:10.1155/2022/3607053)
Supplement: Supplementary Materials — Supplementary Table 1: the 460 DEGs in GSE85871. Supplementary Table 2: the known targets of gastrodin in four drug databases. Supplementary Table 3: 584 gastrodin-related drug targets. Supplementary Table 4: the ADHD-related disease genes. [file 3607053.f1.zip › 3607053.f1/Supplementary Table 4.docx]

| Summary of six disease databases | 267 ADHD disease genes after deletion of duplicates |
| --- | --- |
| HTR1A | HTR1A |
| D2R | D2R |
| D3R | D3R |
| D4R | D4R |
| HTR2A | HTR2A |
| SNDR | SNDR |
| H1R | H1R |
| HTR7 | HTR7 |
| HTR2C | HTR2C |
| HTR1B | HTR1B |
| DAT | DAT |
| NET | NET |
| SERT | SERT |
| ADRA2 | ADRA2 |
| GPR119 | GPR119 |
| GPR55 | GPR55 |
| CB2 | CB2 |
| ADRA1B | ADRA1B |
| TAAR1 | TAAR1 |
| ADRA2A | ADRA2A |
| CHRNA7 | CHRNA7 |
| H3R | H3R |
| CHRM5 | CHRM5 |
| CHRM4 | CHRM4 |
| nAChR | nAChR |
| F10 | F10 |
| CHRNA4 | CHRNA4 |
| ADR | ADR |
| GRIA | GRIA |
| HTR2B | HTR2B |
| TAAR1 | HTR6 |
| HTR6 | SLC6A4 |
| HTR7 | SLC6A2 |
| D2R | KCNQ2 |
| HTR2A | CYP1A2 |
| HTR1A | CYP2C19 |
| HTR6 | CYP2D6 |
| HTR7 | ALB |
| D2R | ORM1 |
| HTR2A | HRH1 |
| HTR1A | ADRA1A |
| SLC6A4 | CYP3A4 |
| SLC6A2 | CYP3A5 |
| KCNQ2 | KCNA1 |
| ADRA2A | ADRA1D |
| CYP1A2 | OPRD1 |
| CYP2C19 | OPRK1 |
| CYP2D6 | NTRK1 |
| ALB | NTRK2 |
| ORM1 | CYP2C9 |
| HRH1 | CYP2B6 |
| HTR2A | CYP2C8 |
| HTR1A | HRH2 |
| ADRA1A | HRH4 |
| CYP3A4 | SIGMAR1 |
| CYP3A5 | HTR1D |
| KCNA1 | OPRM1 |
| ADRA1D | KCNQ3 |
| OPRD1 | UGT2B10 |
| OPRK1 | UGT1A4 |
| NTRK1 | SLC6A3 |
| NTRK2 | CARTPT |
| CYP2C9 | SLC22A3 |
| CYP2B6 | SLC22A5 |
| CYP2C8 | SLC18A2 |
| HRH2 | CYP2A6 |
| HRH4 | MAOB |
| SIGMAR1 | FCGR1A |
| HTR2C | KCNJ3 |
| ADRA1B | CHRNA3 |
| HTR7 | CYP2E1 |
| HTR1D | SLC22A2 |
| OPRM1 | HTR3A |
| HTR1B | ADRA2B |
| HTR6 | ADRA2C |
| KCNQ3 | SLC22A4 |
| UGT2B10 | CYP1A1 |
| UGT1A4 | CES1A1a |
| SLC6A3 | SLC47A1 |
| CARTPT | SLC22A1 |
| TAAR1 | CHRM1 |
| CYP2D6 | CHRM2 |
| SLC22A3 | CHRM3 |
| SLC22A5 | KCND2 |
| SLC18A2 | KCND3 |
| CYP2A6 | CYP2C18 |
| SLC6A2 | CYP3A7 |
| MAOB | KCNH2 |
| SLC6A4 | KCNH1 |
| SLC6A2 | ORM2 |
| CYP2D6 | SLC15A1 |
| SLC6A4 | SLC18A1 |
| CYP2C19 | MAOA |
| ALB | DRD4 |
| FCGR1A | FGD1 |
| KCNJ3 | DRD5 |
| OPRK1 | TPH2 |
| SLC6A3 | TPH1 |
| SLC6A2 | SCN8A |
| CYP2B6 | SNAP25 |
| CYP2D6 | GNB5 |
| ORM1 | CORO1A |
| CHRNA3 | GIT1 |
| CYP2C9 | ANK3 |
| CYP2E1 | ACTL6A |
| SLC22A2 | SLC9A9 |
| HTR3A | CSNK1D |
| ADRA2A | TH |
| CYP2D6 | CES1 |
| ADRA2B | RIC1 |
| ADRA2C | ZNF292 |
| SLC22A3 | SHH |
| SLC22A5 | AUH |
| SLC22A4 | DOCK3 |
| CYP1A2 | NLGN4 |
| CYP3A4 | NRXN1 |
| CYP1A1 | POLG |
| CYP3A5 | CNKSR2 |
| ADRA1A | TCF7L2 |
| ADRA1B | DLD |
| ADRA1D | ADNP |
| ALB | CHD2 |
| SLC6A3 | ERCC4 |
| SLC6A2 | GLRB |
| SLC6A4 | PAFAH1B1 |
| CES1A1a | PPP1R12A |
| ADRA1B | SLC6A8 |
| SLC6A3 | PCDH19 |
| SLC6A2 | YWHAE |
| SLC18A2 | TAAR9 |
| TAAR1 | CRBN |
| CYP2D6 | SAMD9L |
| SLC6A3 | FBN2 |
| ADRA2A | SMAD6 |
| CYP2C19 | ZIC1 |
| CYP3A4 | NKX2-5 |
| ADRA2B | SOX6 |
| SLC47A1 | SLITRK1 |
| SLC22A1 | GUCY2C |
| SLC22A2 | ZMYM3 |
| SLC6A2 | BCORL1 |
| SLC6A4 | TOP3B |
| CYP1A2 | RAI1 |
| CYP2C19 | RTEL1 |
| CYP2D6 | UNC13A |
| HRH1 | RSRC1 |
| ADRA1A | TNRC6B |
| HTR2A | SETBP1 |
| CHRM1 | SRCAP |
| CHRM2 | DYRK1A |
| CHRM3 | MICU2 |
| CHRM4 | TANC2 |
| CHRM5 | ADGRL3 |
| KCND2 | ETFDH |
| KCND3 | PDIA2 |
| ADRA1D | PURA |
| ORM1 | ZDHHC7 |
| SLC22A2 | ZNF134 |
| SLC22A3 | ZNF211 |
| SLC22A4 | WRB |
| CYP3A4 | STRBP |
| CYP2B6 | FXR2 |
| CYP2C18 | ARHGAP12 |
| CYP2E1 | ARSA |
| CYP3A7 | PIGM |
| HTR2C | ARHGEF28 |
| ADRA1B | ELP5 |
| HTR7 | SPSB2 |
| KCNH2 | FARP2 |
| SLC6A3 | CORO7 |
| HTR1A | SENP3 |
| HTR6 | CHURC1 |
| KCNH1 | ZNF565 |
| ALB | RRP7A |
| ORM2 | PYROXD2 |
| CYP2D6 | PEBP4 |
| SLC15A1 | ESYT2 |
| TAAR1 | MIR4278 |
| CYP2D6 | CMTM8 |
| SLC22A3 | OPCML |
| SLC22A5 | CDH23 |
| SLC6A3 | NFIB |
| SLC18A2 | ALDH1L1 |
| TAAR1 | HTT |
| SLC6A4 | TRIB3 |
| SLC6A2 | GRM7 |
| SLC18A1 | MAP2K4 |
| MAOA | GRIN2B |
| MAOB | GGH |
| ADRA2A | DRD1 |
| ADRA2B | DBH |
| ADRA2C | BDNF |
| SLC6A3 | DRD2 |
| SLC6A2 | COMT |
| CES1A1a | ABCB1 |
| HTR1A | DRD1IP |
| SLC6A3 | SYP |
| ADRA1B | RXRB |
| CYP3A4 | RXRA |
| CYP2C19 | RARG |
| CYP1A2 | RARA |
| CYP2B6 | NR4A2 |
| CYP2C9 | NET1 |
| CYP3A5 | HES1 |
| CYP2D6 | RARB |
| MAOB | RXRG |
| CYP2B6 | ADRB2 |
| MAOA | ALDH2 |
| CYP2A6 | ARRB2 |
| CYP3A4 | DRD3 |
| CYP1A2 | FADS1 |
| CYP2C19 | GDNF |
| CYP2C8 | HTR4 |
| CYP2C9 | NT3 |
| CYP2D6 | PNMT |
| SLC6A3 | PRKCG |
| SLC6A2 | NGFB |
| HTR1A | FADS2 |
| CES1A1a | HTR5A |
| DRD4 | SLC6A1 |
| SLC6A3 | SYN3 |
| FGD1 | IL6 |
| DRD5 | KALRN |
| ADRA2A | AGT |
| TPH2 | CTNNA2 |
| HTR1B | CDH13 |
| TPH1 | ASTN2 |
| SCN8A | TNF |
| SNAP25 | A2BP1 |
| SLC6A2 | ABHD13 |
| GNB5 | ADAMTS2 |
| CORO1A | AMOTL1 |
| GIT1 | ATP8B1 |
| ANK3 | C12orf28 |
| ACTL6A | C16orf46 |
| SLC9A9 | C5orf15 |
| CSNK1D | CWC15 |
| TH | DHODH |
| CES1 | ETV3 |
| RIC1 | ETV3L |
| ZNF292 | FLJ31818 |
| SHH | FLJ39061 |
| AUH | FZD7 |
| DOCK3 | GLT25D2 |
| HTR2B | GPR85 |
| NLGN4 | GSX1 |
| NRXN1 | JMJD2D |
| POLG | KIAA0174 |
| MAOA | KIF6 |
| CNKSR2 | KIRREL3 |
| TCF7L2 | LIG4 |
| DLD | LOC729257 |
| ADNP | PAWR |
| CHD2 | PDX1 |
| ERCC4 | PITRM1 |
| GLRB | PIWIL4 |
| PAFAH1B1 | PKD1L2 |
| PPP1R12A | PKD1L3 |
| SLC6A8 | PTPRD |
| PCDH19 | RGL1 |
| YWHAE | SPATA8 |
| TAAR9 | SULF2 |
| CRBN | YWHAZ |
| SAMD9L | STS |
| FBN2 | NLGN3 |
| SMAD6 | NSDHL |
| ZIC1 | CHRNB2 |
| NKX2-5 |  |
| SOX6 |  |
| SLITRK1 |  |
| GUCY2C |  |
| ZMYM3 |  |
| BCORL1 |  |
| TOP3B |  |
| RAI1 |  |
| RTEL1 |  |
| UNC13A |  |
| RSRC1 |  |
| TNRC6B |  |
| SETBP1 |  |
| SRCAP |  |
| DYRK1A |  |
| MICU2 |  |
| TANC2 |  |
| ADGRL3 |  |
| ETFDH |  |
| PDIA2 |  |
| PURA |  |
| ZDHHC7 |  |
| ZNF134 |  |
| ZNF211 |  |
| WRB |  |
| STRBP |  |
| FXR2 |  |
| ARHGAP12 |  |
| ARSA |  |
| PIGM |  |
| ARHGEF28 |  |
| ELP5 |  |
| SPSB2 |  |
| FARP2 |  |
| CORO7 |  |
| SENP3 |  |
| CHURC1 |  |
| ZNF565 |  |
| RRP7A |  |
| PYROXD2 |  |
| PEBP4 |  |
| ESYT2 |  |
| MIR4278 |  |
| CMTM8 |  |
| OPCML |  |
| CDH23 |  |
| NFIB |  |
| ALDH1L1 |  |
| HTT |  |
| TRIB3 |  |
| GRM7 |  |
| SNAP25 |  |
| ADGRL3 |  |
| MAP2K4 |  |
| GRIN2B |  |
| TH |  |
| HTR1B |  |
| CHRNA4 |  |
| TPH2 |  |
| GGH |  |
| DRD4 |  |
| DRD1 |  |
| DBH |  |
| SLC6A2 |  |
| ADRA2A |  |
| SLC6A3 |  |
| CES1 |  |
| BDNF |  |
| SLC6A4 |  |
| DRD2 |  |
| COMT |  |
| ABCB1 |  |
| CYP2D6 |  |
| DRD5 |  |
| DRD4 |  |
| DRD2 |  |
| DRD1 |  |
| DRD1IP |  |
| DOCK3 |  |
| CHRNA4 |  |
| ADRA2C |  |
| ADRA2A |  |
| SYP |  |
| SNAP25 |  |
| SLC6A4 |  |
| SLC6A3 |  |
| SLC6A2 |  |
| RXRB |  |
| RXRA |  |
| RARG |  |
| RARA |  |
| NR4A2 |  |
| NET1 |  |
| HTR2C |  |
| HTR2A |  |
| HTR1B |  |
| TPH1 |  |
| TPH2 |  |
| BDNF |  |
| COMT |  |
| DBH |  |
| HES1 |  |
| RARB |  |
| MAOA |  |
| RXRG |  |
| ADRB2 |  |
| ALDH2 |  |
| ARRB2 |  |
| DRD3 |  |
| FADS1 |  |
| GDNF |  |
| GRIN2B |  |
| HTR1D |  |
| HTR4 |  |
| HTR6 |  |
| MAOB |  |
| NT3 |  |
| PNMT |  |
| PRKCG |  |
| NGFB |  |
| FADS2 |  |
| HTR5A |  |
| SLC6A1 |  |
| SYN3 |  |
| IL6 |  |
| KALRN |  |
| AGT |  |
| CTNNA2 |  |
| CES1 |  |
| CDH13 |  |
| ASTN2 |  |
| TNF |  |
| A2BP1 |  |
| ABHD13 |  |
| ADAMTS2 |  |
| AMOTL1 |  |
| ATP8B1 |  |
| C12orf28 |  |
| C16orf46 |  |
| C5orf15 |  |
| CWC15 |  |
| DHODH |  |
| ETV3 |  |
| ETV3L |  |
| FLJ31818 |  |
| FLJ39061 |  |
| FZD7 |  |
| GLT25D2 |  |
| GPR85 |  |
| GSX1 |  |
| JMJD2D |  |
| KIAA0174 |  |
| KIF6 |  |
| KIRREL3 |  |
| LIG4 |  |
| LOC729257 |  |
| PAWR |  |
| PDX1 |  |
| PITRM1 |  |
| PIWIL4 |  |
| PKD1L2 |  |
| PKD1L3 |  |
| PTPRD |  |
| RGL1 |  |
| SPATA8 |  |
| SULF2 |  |
| YWHAZ |  |
| STS |  |
| CHRNA7 |  |
| NLGN3 |  |
| DRD4 |  |
| NSDHL |  |
| CHRNB2 |  |
